# Response shift results of quantitative research using patient-reported outcome measures: a meta-regression analysis

**DOI:** 10.1007/s11136-024-03867-x

**Published:** 2024-12-09

**Authors:** Richard Sawatzky, Mathilde G. E. Verdam, Yseulys Dubuy, Tolulope T. Sajobi, Lara Russell, Oluwagbohunmi A. Awosoga, Ayoola Ademola, Jan R. Böhnke, Oluwaseyi Lawal, Anita Brobbey, Amélie Anota, Lisa M. Lix, Mirjam A. G. Sprangers, Véronique Sébille, the Response Shift – in Sync Working Group

**Affiliations:** 1 School of Nursing, Trinity Western University, 7600 Glover Road, Langley, BC V2Y 1Y1 Canada; 2 Centre for Advancing Health Outcomes, Providence Health Care Research Institute, Vancouver, Canada; 3 Institute of Health and Care Sciences, and Centre for Person-Centred Care (GPCC), Sahlgrenska Academy, University of Gothenburg, Gothenburg, Sweden; 4 Medical Psychology, Amsterdam UMC Location University of Amsterdam, Amsterdam, the Netherlands; 5 Department of Methodology and Statistics, Institute of Psychology, Leiden University, Leiden, The Netherlands; 6 Nantes Université, Université de Tours, CHU Nantes, INSERM, MethodS in Patient-Centered Outcomes and HEalth ResEarch, SPHERE, Nantes, F-44000 France; 7 Department of Community Health Sciences, University of Calgary, Calgary, Canada; 8 Faculty of Health Sciences, University of Lethbridge, Lethbridge, Canada; 9 School of Health Sciences, University of Dundee, Dundee, UK; 10 Department of Clinical Research and Innovation, Centre Léon Bérard, Lyon, France; 11 Department of Community Health Sciences, University of Manitoba, Winnipeg, Canada; 12 Amsterdam Public Health, Mental Health, Amsterdam, the Netherlands

**Keywords:** Response shift, Patient reported outcomes, Meta-regression, Effects, Effect sizes

## Abstract

**Purpose:**

Our objectives were to identify characteristics of response shift studies using patient-reported outcomes (PROMs) that explain variability in (1) the detection and (2) the magnitude of response shift effects.

**Methods:**

We conducted a systematic review of quantitative studies published before June 2023. First, two-level multivariable logistic regression models (effect- and sample-levels) were used to explain variability in the probability of finding a response shift effect. Second, variability in effect sizes (standardized mean differences) was investigated with 3-level meta-regression models (participant-, effect- and sample-levels). Explanatory variables identified via the purposeful selection methodology included response shift method and type, and population-, study design-, PROM- and study-quality characteristics.

**Results:**

First, logistic regression analysis of 5597 effects from 206 samples in 171 studies identified variables explaining 41.5% of the *effect-level* variance, while no variables explained *sample-level* variance. The average probability of response shift detection is 0.20 (95% CI: 0.17-0.28). Variation in detection was predominantly explained by response shift methods and type (recalibration vs. reprioritization/reconceptualization). Second, effect sizes were analyzed for 769 effects from 114 samples and 96 studies based on the then-test and structural equation modeling methods. Meta-regression analysis identified variables explaining 11.6% of the effect-level variance and 26.4% of the sample-level variance, with an average effect size of 0.30 (95% CI: 0.26-0.34).

**Conclusion:**

Response shift detection is influenced by study design and methods. Insights into the variables explaining response shift effects can be used to interpret results of other comparable studies using PROMs and inform the design of future response shift studies.

**Supplementary Information:**

The online version contains supplementary material available at 10.1007/s11136-024-03867-x.

## Background

Patient-reported outcome measures (PROMs) are increasingly used in empirical studies and clinical practice to assess, among other things, the effectiveness of healthcare interventions and to monitor patients’ quality of life (QOL) over time. However, longitudinal measurements of patient-reported outcomes can be affected by response shift. Schwartz & Sprangers [1, 2] defined response shift as a change in the meaning of one’s self-evaluation of a target construct as a result of a change in one’s internal standards of measurement (recalibration), a change in the importance of component domains constituting the target construct (reprioritization), or a redefinition of the target construct (reconceptualization). When response shift occurs, PROM results will not have the same meaning at different points in time. Consequently, the change in observed PROM scores will not accurately reflect change in the construct that the PROM intends to measure (a.k.a. change in the “target construct”). This difference between “observed” and “target” change has been operationalized as a response shift effect. Whereas response shift may invalidate comparisons of PROM results over time when it is not taken into account, response shift is also viewed as meaningful information that provides insight into how patients accommodate health changes [1, 2].

Over the past decades, many studies have been conducted to investigate occurrences and magnitudes of response shift effects. Two systematic reviews on the detection of response shift have been published, encompassing 101 [3] and 107 [4] studies with 51 overlapping. Previous systematic reviews on the magnitudes of response shift effects include meta-analyses of: (a) studies published up to 2005 that examined response shift based on the then-test (one of the most commonly used response shift detection methods that involves asking respondents to retrospectively re-evaluate their baseline functioning at posttest. The comparison of scores on the baseline measure and the then-test provides an indication of response shift and its magnitude) [5], (b) studies published up to 2016 on people with an orthopedic condition [6], and (c) studies published up to 2018 on people with cancer [7]. Hence, these reviews were restricted in the outcome (i.e., detection or magnitude of response shift), the method, or the target population. We previously conducted a descriptive systematic review of all quantitative studies published before 2021 that investigated response shift using PROMs and described distributions of response shift detection and, where possible, effect sizes [8]. The results of this descriptive review provided insight into *how* the number and magnitude of response shift effects vary across diverse studies employing different response shift methods, populations, research designs, and PROMs.

The next important aim is to gain insight into *why* response shift results differ, by investigating relevant variables that are associated with and explain variability in response shift results. The current meta-regression analysis builds on the previous descriptive review and aims to identify response shift methods, population characteristics, design characteristics, PROMs, and study quality characteristics that explain variability in (1) the detection of response shift effects and (2) the magnitude of response shift effects (i.e., standardized mean differences). This latter objective was only investigated for studies using the then-test and/or structural equation modeling (SEM) methods that enable the calculation of a standardized effect size for measuring the difference between means (Cohen’s *d*). This work is part of the Response Shift – in Sync Working Group initiative that aims to synthesize the work on response shift to date [9–13].

## Methods

We conducted a systematic review and meta-regression analysis (registered retrospectively in INPLASY at the time of data analysis: #202290033) [14] following guidelines by Cooper, Hedge, and Valentine [15] and used the PRISMA statement as a guide for reporting the results [16].

### Search strategy and eligibility criteria

**Fig. 1:**
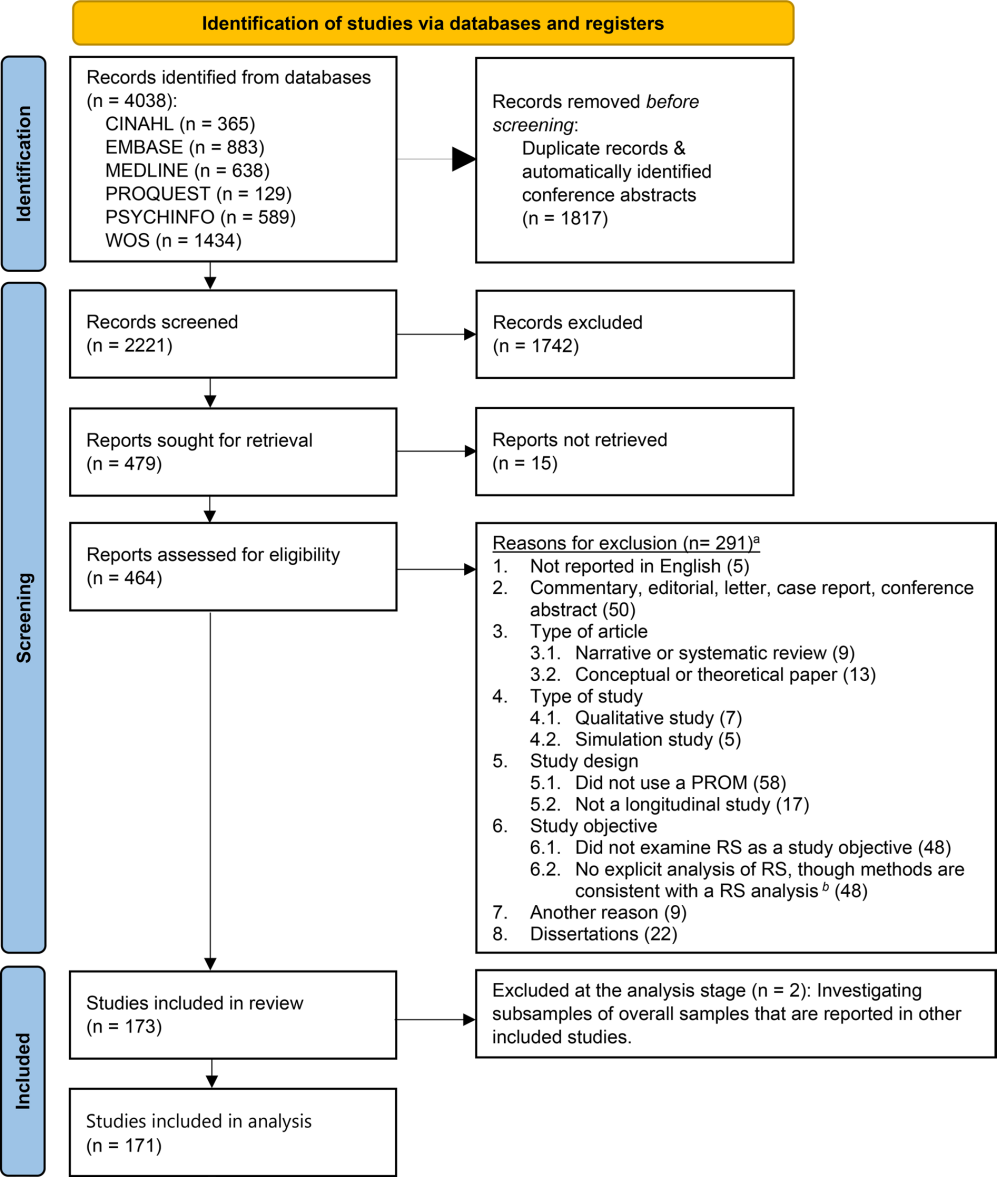
PRISMA flow diagram. *Notes*: RS = Response shift. PROM = Patient-reported outcome measure. HRQOL = Health-related quality of life. ^a)^Reasons are ranked by the first identified reason for exclusion. ^b)^ When method- and result- sections were not explicitly related to RS analyses, these studies were excluded (e.g., longitudinal measurement invariance studies were excluded if they did not explicitly follow a recognized SEM method for response shift detection (e.g., Oort’s SEM approach)

We aimed to include all longitudinal quantitative studies that examined response shift using a PROM. The search strategy, eligibility criteria, study selection and data extraction procedures, and the use of the EPPI Reviewer Platform [17] were identical to those described in the publication of our previous descriptive systematic review [8]. Since the previous review contained articles published before 2021, we have updated the literature search to May 2023, following the same procedures. The additional studies were randomly assigned for independent screening of titles and abstracts by two team members (RS, VS, MAGS). The full text was retrieved for all citations identified as potentially relevant and were screened randomly by two of the same team members. Disagreements were reconciled via consensus. The updated search led to the inclusion of 23 studies, in addition to the 150 studies from the previous systematic review [8], resulting in 173 studies that fulfilled the eligibility criteria. Exclusion criteria were sequentially applied in the order shown in PRISMA flow diagram (see Fig. 1).

### Data extraction

We used the data extracted from the 150 studies included in the previous systematic review [8] and completed data extraction (by one of two team members: MV and RS) for the additional included studies following the same procedures (see [8] for details). Data were extracted for the following explanatory variables, which were also included in the original systematic review (see corresponding categories in Table 1): population-, study design-, and PROM characteristics, and response shift type and methods. Additionally, data were extracted for the following study quality indicators, which were included as study quality control variables in the multivariable analyses: primary/secondary analysis, hypothesis stated (yes/no), missing data reported (yes/no), response shift explanation provided (whether authors explored relations between response shifts and other explanatory variables) (yes/no). The primary/secondary analysis variable was part of design characteristics in the original descriptive review [8], whereas the latter three variables are new.

**Table 1:** Results of the 2-level multivariable logistic regression model explaining variability in response shift detection

Explanatory/control variables	*N* Studies	*N* Samples	*N* Effects	Marginal probability of RS detection (95% CI)^a^	Pratt Index (% *R*^2^)^b^
**Sample-level variables (** ***R*** ^**2**^ ** = 7.2%)**					
***Population characteristics***					
**Sex**					
Mixed (ref)	144	167	4705		
Only female	13	14	517		n/a
Only male	10	13	161		n/a
Other/unknown	6	12	214		n/a
**Age**					
Mostly adults (ref)	120	146	4033		
Mostly older adults	34	37	702		n/a
Mostly children/adolescents	8	9	225		n/a
Other/Unknown	10	14	637		n/a
**Medical condition**					
Yes: Cancer (ref)	48	51	1707	0.23 (0.19–0.33)	n/a
Yes: Orthopedic	10	11	96	0.20 (0.10–0.38)	-1.8
Yes: Stroke	10	11	472	0.16 (0.09–0.26)	19.4
Yes: Mental health	14	24	1118	0.19 (0.12–0.34)	0.4
Yes: Other	81	96	2081	0.19 (0.16–0.28)	2.1
No	10	13	123	0.17 (0.10–0.40)	11.2
**Intervention**					
No/Unclear (ref)	55	72	1814	0.17 (0.13–0.25)	n/a
Yes: Medical	79	82	2361	0.22 (0.18–0.31)	39.2
Yes: Psychological	27	39	1298	0.21 (0.14–0.34)	1.3
Yes: Other/unspecified	10	13	124	0.27 (0.16–0.51)	28.2
**Effect-level variables (*R***^**2**^ **= 41.5%)**					
***Study design characteristics***					
**Design**					
Observational (ref)	138	159	4402	0.21 (0.19–0.30)	n/a
Experimental	33	47	1195	0.16 (0.11–0.24)	3.4
**Sample size**					
Q1 (< 57)	41	50	352	0.12 (0.08–0.23)	-0.1
Q2 (57–254)	86	93	1823	0.20 (0.18–0.28)	0.6
Q3 (255–410)	34	32	1296	0.24 (0.21–0.35)	3.2
Q4 (> 411) (ref)	38	41	2126	0.19 (0.13–0.28)	n/a
**Time period classification**					
< 1 month	19	24	661	0.16 (0.13–0.25)	1.6
1–6 months (ref)	97	114	3090	0.20 (0.16–0.29)	n/a
> 6 months − 12 months	44	51	976	0.25 (0.19–0.35)	0.7
> 12 months	25	35	367	0.27 (0.22–0.40)	3.2
Not reported	13	14	503	0.15 (0.08–0.34)	1.5
***PROM characteristics***					
**PROM type**					
Generic PROMs (ref)	82	97	2043	0.20 (0.17–0.30)	n/a
Disease-specific PROMs	65	73	1847	0.18 (0.14–0.26)	0.2
Individualized/other PROMs	63	75	1707	0.23 (0.17–0.33)	-1.0
**PROM domain**					
General health/QOL (ref)	109	122	710	0.25 (0.21–0.34)	n/a
Physical	106	132	1949	0.21 (0.18–0.28)	0.0
Psychological	100	120	1723	0.18 (0.15–0.26)	3.5
Social	62	73	548	0.19 (0.15–0.28)	-0.2
Pain	53	62	244	0.27 (0.20–0.36)	0.3
Other	32	33	423	0.15 (0.12–0.24)	1.4
***Response shift type and method***					
**Response shift type**					
Recalibration (ref)	138	170	2153	0.29 (0.25–0.37)	n/a
Reprioritization/reconceptualization	104	130	3444	0.14 (0.12–0.22)	22.1
**Response shift method** ^**c**^					
Then-Test (ref)	82	94	708	0.30 (0.19–0.47)	
Latent variable method^d^	58	81	4245	0.14 (0.11–0.21)	30.5
Regression method^e^	26	31	492	0.50 (0.33–0.71)	10.2
Other^e^	30	31	152	0.44 (0.27–0.64)	1.8
***Study quality control variables***					
**Primary data analysis**					
No (ref)	75	102	4032	0.17 (0.14–0.25)	n/a
Yes	96	107	1565	0.27 (0.19–0.39)	12.6
**666Hypothesis about response shift**					
No (ref)	96	110	3772	0.22 (0.19–0.30)	n/a
Yes	75	101	1825	0.17 (0.14–0.23)	0.7
**Explanation of response shift**					
No (ref)	79	75	2619	0.17 (0.15–0.25)	n/a
Yes	92	136	2978	0.23 (0.18–0.32)	2.1
**Missing data reported**					
No (ref)	137	172	4795	0.19 (0.16–0.26)	n/a
Yes	34	36	802	0.26 (0.18–0.45)	1.9

*Notes*: N = the corresponding # of studies, # of samples, or # of response shift effects. The # of studies and # of samples do not add up to their respective totals or subtotals (bolded rows) because multiple categories for each explanatory variable could apply to the same study or sample. n/a = not available for reference categories or because the variable was not retained in the multivariable model. ref = reference category of dummy-coded variables. Marginal probabilities were calculated by applying the marginal standardization approach to the results of the 2-level multivariable logistic regression analysis, with bootstrapped confidence intervals (see methods section for details). ^b)^The percentages do not add up exactly to 100% for each level because due to rounding of parameter estimates. Negative percentages may also be due to rounding of parameter estimates or may indicate the possibility of variable suppression. ^c)^See Table 1 of Sawatzky et al., 2023 [8] for detailed descriptions. ^d)^Includes structural equation modeling, item response theory and Rasch measurement theory methods. ^e)^Includes regression methods with and without classification. ^f)^Includes design-based methods other than the then-test (individualized methods, ideal scale approach, appraisal, change in importance ratings) as well as study-specific methods

The outcome variables were:

1. **Detection of response shift**: Evidence about whether response shift was detected (yes/no) at the level of individual response-shift effects. This categorization was based on the conclusions of the authors of the primary studies regarding the evidence of response shift, where these conclusions may have been based on different grounds (e.g., different studies adopted different alpha levels to determine the statistical significance of the effects).
2. **Magnitude of response shift**: Effect sizes based on standardized mean differences of individual response shift effects for the then-test and SEM methods. The magnitude of each response shift effect was based on reported statistical information, where possible. Table 1 in Sawatzky et al. [8] includes a detailed description of effect size calculation for both methods. Standardized mean differences (Cohen’s *d*) were calculated based on the difference between mean baseline ($\:{\stackrel{-}{X}}_{1})$ and follow-up (then-test) ($\:{\stackrel{-}{X}}_{2})$ scores as follows: Cohen’s *d* = $\:\frac{{\stackrel{-}{X}}_{1}-{\stackrel{̿}{X}}_{2}}{SD}$ (where SD = standard deviation). The SD was calculated based on the following hierarchy: (i) the SD of the difference in means; (ii) the pooled SD; or (iii) the SD of the baseline measure. For some studies that did not provide the information required for these calculations we first had to transform medians, interquartile ranges (IQR), confidence intervals (CI), or *t* statistics into corresponding means and standard deviations [15, 18]. For SEM, Cohen’s *d* for response shift effects were based on parameter estimates of models that adjust for a lack of longitudinal measurement invariance (for more information see [19]). We used reported effect sizes, if provided, when insufficient information was available to calculate effect sizes. We included all response shift effects for which an effect size could be calculated or was reported, including response shift effects that were not statistically significant. All effect sizes were converted to absolute values because interpretation of the direction of effect is lost due to the heterogeneity of studies (i.e., expected change can be both positive and negative).

### Statistical analyses

**Fig. 2:**
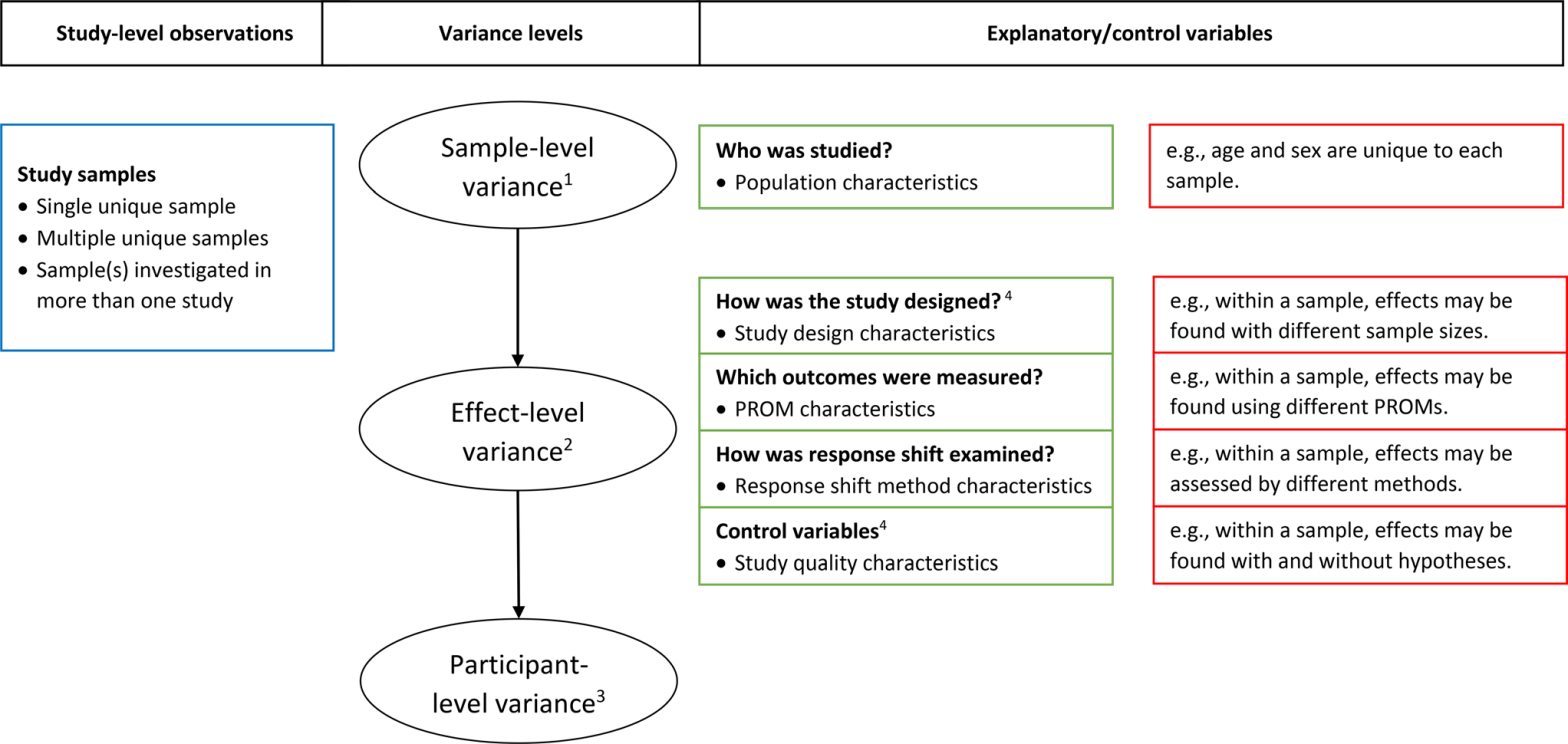
Relationship between study-level observations, variance levels of the modeling procedure, and explanatory/control variables. *Notes*: ^1^The samples are assumed to be independent. ^2^Accounts for instances where multiple effects are based on the same sample. ^3^Accounts for individual-level variation among the participants. These are based on the variances (and sample sizes) of the primary studies. ^4^ The study design and study quality characteristics are effect-level variables because some samples are investigated in more than one study and therefore there may be variability in these characteristics within samples

The current meta-regression analysis focuses on explaining variability in the detection and magnitude of response shift effects, using population-, study design-, and PROM-characteristics, and response shift type and methods as explanatory variables, and as a last step, study quality indicators as control variables (see explanation in the risk of bias section below). Figure 2 provides an overview of how the study-level observations are related to the variance levels of the modeling procedure and the explanatory/control variables. We used a multilevel framework for our analyses and included (unique) sample identifications to take into account that response shift effects are investigated in the same sample [20]. Some of the samples were included in multiple studies (e.g., for secondary analyses with another response shift method), whereas other studies included multiple (unique) samples; for example, when response shift effects were investigated in a treatment and control group. We used sample identification as the appropriate nesting variable because dependency between response shift effects will most likely occur when they are investigated in the same group of participants, rather than in the same study. When samples were overlapping, only the overall sample was included (i.e., subsamples were excluded). See Data Availability and Code Availability sections to access the data files and syntax for all analyses.

Variability in the detection of response shift was investigated with 2-level (effect- and sample-levels) multivariable logistic regression models [20]. The models do not include participant-level sampling variance because information about the detection of response shift (yes/no) was only available at the level of the study sample (based on the conclusions made by the authors of the primary studies) and not at the level of participants within a sample, for all but a few studies. We calculated marginal probabilities of response shift detection for each level of the explanatory variables using the marginal standardization approach [21, 22]. Marginal standardization estimates take into account that some categories of the explanatory variable have few observations and are appropriate for making inferences about the overall population. Methods for bootstrapping of clustered data were applied to obtain 95% CIs [23]. To provide information about the relative importance of individual explanatory variables in explaining variability in response shift detection, we calculated the Pratt Index based on the results of the final multilevel model [24, 25]. The Pratt Index shows the proportion of the explained variance attributable to each explanatory variable. The Mplus software (version 8.10) was used to obtain the standardized parameter estimates and correlations and to calculate the explained variance based on the *R*^2^ of the latent variable underlying the binary criterion model, as is further explained by Bosker and Snijders (2011) [26].

Variability in the magnitude of response shift was investigated using 3-level meta-regression models to account for dependencies between effects from the same sample [27, 28]. The analyses account for participant sampling variance (level 1), as this is inherent in the calculation of Cohen’s d effect size, as well as effect- and sample-level variances (levels 2 and 3). The variance decomposition and variance explained (*R*^2^) by the explanatory variables at levels 2 and 3 were calculated as suggested by Cheung [27]. The statistical analyses were performed using the Mplus software (version 8.10) and packages lme4 (v1.1-34) [29] and metaSEM (v1.3.1) [30] in R [31].

We followed Hosmer and Lemeshow’s purposeful selection procedure [32] to identify variables that explain variability in the detection of response shift effects (based on the 2-level multivariable logistic models) and magnitude of response shift effects (based on the 3-level meta-regression models). This procedure strikes a balance between including important variables and maintaining statistical rigor in model building while ensuring that the selected model is clinically meaningful and statistically valid. The procedure begins by including variables that are deemed clinically or theoretically important and uses a combination of statistical and substantive criteria to arrive at a final selection of explanatory variables. In the first step, univariate models for each explanatory variable (i.e., response shift methods, population-, study design-, and PROM-characteristics) were tested for statistical significance using the criterion of α = 0.25. A conservative α was chosen as a screening criterion for initial variable selection to ensure the selection of potentially important variables. In step 2, a multivariable model was fitted with all explanatory variables selected in step 1. Using an iterative approach, each explanatory variable was removed from the multivariable model one-by-one, and the impact of this removal on the multivariable model was investigated in terms of statistical significance (*p* < 0.10) and confounding (a change of > 20% in one or more of the regression coefficients of the other variables in the model). Each explanatory variable that did not meet either criterion was excluded from the multivariable model. This iterative procedure was continued until all explanatory variables in the multivariable model met at least one of the two selection criteria. In step 3 the variables that were not selected in step 1 (*p* > 0.25) were added to the final multivariable model from step 2 to re-evaluate their contribution and were retained if *p* < 0.10. The control variables were subsequently added (without further selection) to control for the potential effects of study quality characteristics. Possible interaction effects between the explanatory variables were not considered.

### Risk of bias

We did not perform a formal assessment of the methodological quality or risk of bias of individual studies. We do not believe that such a formal assessment could be conducted in an unambiguous and meaningful way, given the heterogeneity of the included studies, the predominance of observational designs for which risk of bias assessments are less straightforward, the inconsistent and incomplete reporting of quality indicators, and the current state of response shift research where quality requirements are contextually dependent and often unknown. For example, we do not know which catalytic event with which intensity and duration is needed or which follow-up period is of sufficient duration to be certain that a response shift would occur. However, for each study, we extracted several control variables that could be considered indications of study quality (i.e., whether a response shift hypothesis was formulated, whether primary or secondary analyses were conducted, whether missing data were reported, and whether a response shift explanation was provided). We adopted a post-hoc, empirical approach by including these control variables together with other explanatory variables in the meta-regression analysis [33].

## Results

### Studies, samples, and response shift effects

Of the 4038 records screened, 173 studies fulfilled the eligibility criteria (see Fig. 1. A reference list of included studies is provided in the Supplementary Appendix). We identified a total of 6154 response shift effects, of which 557 were excluded from analyses because they were from 101 subsamples of overall samples reported in the same or another study. Two studies that only investigated subsamples of overall samples investigated in another study were also excluded. The analysis therefore included 5597 effects from 206 samples that were investigated in 171 studies, of which 148 studies were unrelated to any of the other studies and 23 studies involved samples that were also analyzed in other studies. Of the included effects, 4577 effects were from 197 independent samples that were each analyzed in only one study, and 1020 effects were from 9 samples investigated in more than one study. Of the 5597 effects, 812 (14.5%) were identified as a response shift, based on criteria defined by the authors.

### Explaining variability in detection of response shift

**Fig. 3:**
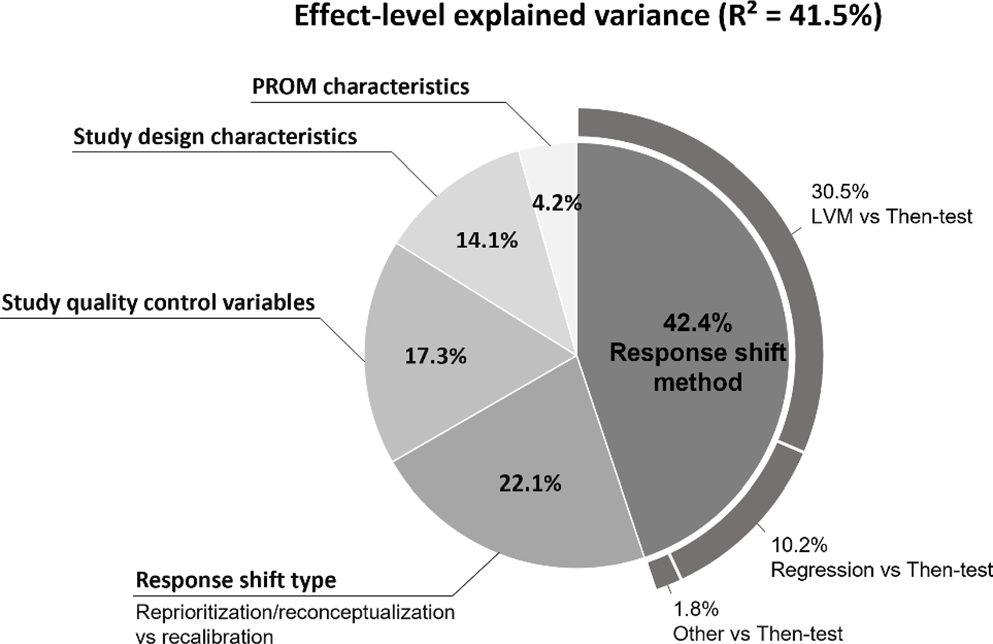
Partitioning of explained variation in detection of response shift effects. *Notes:* The size of each slice and its corresponding value indicates the percentage of *effect-level* explained variance. The explained variance attributable to the different response shift methods, when compared to the then-test method, are partitioned out in the outer ring. The percentages do not add up exactly to their corresponding totals due to rounding errors of parameter estimates. *Sample-level* explained variance is not depicted because the sample-level variables (medical conditions and interventions) did not statistically significantly explain any of the sample-level variance (*R*^2^ = 7.2%, *p* = 0.19)

Following the purposeful selection procedure, sex and age were the only explanatory variables that did not meet the criteria for inclusion (results of the purposeful variable selection procedure are reported in Supplementary Table S1, with corresponding parameter estimates of the final model provided in Supplementary Table S2). The explanatory variables in the final 2-level multivariable logistic regression model explained 41.5% (95% CI: 0.34–0.49) of the effect-level variance in response shift detection (see Table 1). As shown in Fig. 3, most of the effect-level explained variance was attributable to differences in response shift methods (42.4%), in particular, the then-test method versus a latent variable method (30.5% of the effect-level explained variance). Moreover, response shift type (recalibration versus other) accounted for 22.1% of the effect-level variance. The study quality control variables accounted for 17.3% of the effect-level explained variance, where the distinction of primary versus secondary data analysis was the most important (accounting for 12.6% of the effect-level explained variance; not shown in Fig. 3). Study design characteristics (including observational versus experimental design, sample size, and time period classification) accounted for 14.1% of the effect-level explained variance, whereas PROM characteristics (types and domains) accounted for only 4.2%. At the sample level, the two retained explanatory variables (types of interventions and medical conditions) explained only 7.2% (95% CI: -0.04-0.18) of sample-level variance with a *p*-value of 0.19.

The overall marginal probability (P) of detecting response shift, while taking into account dependencies between effects investigated in the same sample (see Table 1), was 0.20 (95% CI: 0.17–0.28). The three smallest marginal probabilities were for effects based on small sample sizes of < 57 (*P* = 0.12, based on 352 effects from 50 samples), latent variable methods (*P* = 0.14, based on 4245 effects from 81 samples), and reprioritization/reconceptualization response shift (*P* = 0.14, based on 3444 effects from 130 sample). The three largest marginal probabilities were for regression methods (*P* = 0.50, based on 492 effects from 31 samples), other response shift methods (i.e., not then-test, latent variable methods, or regression methods) (*P* = 0.44, based on 152 effects from 31 samples), and the then-test (*P* = 0.30, based on 708 effects from 94 samples).

### Explaining variability in magnitude of responses shift

Information about effect sizes was available for 769 effects from 116 samples and 97 studies using the then-test (637 effects from 81 samples and 72 studies) or SEM (132 effects from 39 samples and 27 studies) (see Table 2). Following the purposeful selection procedure, sex and sample size did not meet the criterion for inclusion. All other explanatory variables were retained in the final model. Response shift method (then-test vs. SEM) and the four study quality control variables were retained because they were of theoretical interest, but these did not show any statistically significant relations with effect-size magnitude (results of the purposeful variable selection procedure are reported in Supplementary Table S3, with corresponding parameter estimates of the final model provided in Supplementary Table S4).

**Table 2:** Results of the 3-level meta-regression model explaining variability in response shift effect sizes

Explanatory/control variables	*N* Studies	*N* Samples	*N* Effects	Marginal mean effect size (95% CI)
**Sample-level variables (** ***R*** ^**2**^ ** = 26.4%)**				
**Population characteristics**				
**Sex**				
Mixed	77	88	466	n/a
Only female	9	8	215	n/a
Only male	8	11	69	n/a
Other/unknown	4	9	19	n/a
**Age**				
Mostly Adults	65	78	562	0.31 (0.27–0.36)
Mostly Older adults	21	23	121	0.30 (0.22–0.38)
Mostly Children/adolescents	5	6	26	0.10 (-0.06–0.26)
Other/Unknown	6	9	60	0.30 (0.16–0.44)
**Medical condition**				
Yes: Cancer	34	35	429	0.31 (0.24–0.37)
Yes: Orthopedic	9	10	79	0.37 (0.25–0.48)
Yes: Stroke	4	6	14	0.16 (-0.02–0.33)
Yes: Mental health	4	8	8	0.43 (0.22–0.64)
Yes: Other	42	51	214	0.26 (0.20–0.32)
No	6	6	25	0.41 (0.26–0.57)
**Intervention**				
No/unclear	23	28	122	0.26 (0.18–0.34)
Yes: Medical	55	58	528	0.28 (0.23–0.33)
Yes: Psychological	14	24	66	0.38 (0.28–0.48)
Yes: Other/unspecified	5	6	53	0.32 (0.17–0.47)
**Effect-level variables (*R***^**2**^ **= 11.6%)**				
***Study design characteristics***				
**Design**				
Observational	81	89	618	0.29 (0.25–0.34)
Experimental	16	27	151	0.34 (0.25–0.43)
**Sample size**				
Q1 (< 57)	31	38	177	n/a
Q2 (57–254)	53	57	325	n/a
Q3 (255–410)	18	16	200	n/a
Q4 (> 411)	11	10	67	n/a
Not reported	0	0	0	n/a
**Time period classification**				
< 1 month	13	16	102	0.27 (0.22–0.34)
1–6 months	66	82	470	0.29 (0.25–0.34)
> 6 months − 12 months	21	20	79	0.31 (0.23–0.39)
> 12 months	11	14	50	0.45 (0.35–0.55)
Not reported	5	6	68	0.25 (0.17–0.33)
***PROM characteristics***				
**PROM type**				
Generic PROMs	46	55	241	0.24 (0.19–0.30)
Disease-specific PROMs	44	45	390	0.35 (0.29–0.40)
Individualized/other PROMs	28	37	138	0.26 (0.20–0.32)
**PROM domain**				
General health/QOL	56	60	113	0.31 (0.26–0.36)
Physical	62	74	311	0.28 (0.24–0.33)
Psychological	51	54	152	0.27 (0.22–0.32)
Social	34	34	68	0.32 (0.26–0.38)
Pain	29	32	63	0.39 (0.33–0.44)
Other	21	21	62	0.31 (0.25–0.37)
***Response shift type and method***				
**Response shift type**				
Recalibration	95	106	732	0.30 (0.26–0.34)
Reprioritization/reconceptualization	14	20	37	0.22 (0.14–0.30)
**Response shift method**				
Then-Test	72	81	637	0.29 (0.25–0.33)
Structural equation modeling	27	39	132	0.34 (0.27–0.41)
***Study quality control variables***				
**Primary data analysis**				
No	25	35	136	0.27 (0.19–0.34)
Yes	72	82	633	0.30 (0.26–0.35)
**Hypothesis about response shift**				
No	53	61	470	0.29 (0.24–0.34)
Yes	44	58	299	0.31 (0.26–0.36)
**Explanation of response shift**				
No	44	42	397	0.29 (0.24–0.34)
Yes	53	76	372	0.32 (0.28–0.36)
**Missing data reported**				
No	81	102	726	0.30 (0.26–0.34)
Yes	16	15	43	0.30 (0.20–0.39)

*Notes*: N = the corresponding # of studies, # of samples, or # of response shift effects. The # of studies and # of samples do not add up to their respective totals or subtotals (bolded rows) because multiple categories for each explanatory variable could apply to the same study or sample. n/a = not available because the variable was not retained in the multivariable model

Of the total variance in magnitude of response shift effects (0.053), 55% was due to variance at the *sample-level* (i.e., estimated sample-level variance = 0.029) and 37% was due to variance at the *effect-*level (i.e., estimated effect-level variance = 0.019). The explanatory variables in the final meta-regression model explained 11.6% of the effect-level variance and 26.4% of the sample-level variance in the response shift effect sizes. The average estimated effect size for response shift effects was 0.30 (95% CI: 0.26–0.34). All but two of the marginal mean effect sizes for the explanatory/control variables were of small to moderate magnitude (based on Cohen’s [34] conventions), ranging from 0.22 to 0.45 (see Table 2). The two exceptions were for mostly children/adolescents (*d* = 0.10, based on 26 effects from 6 samples) and for stroke (*d* = 0.16, based on 14 effects from 6 samples). Of the remaining marginal effect sizes, the smallest effect size of 0.22 was for reprioritization/reconceptualization response shift (based on 37 effects from 20 samples). The largest effect size was for the time-period > 12 months (*d* = 0.45, based on 50 effects from 14 samples).

## Discussion

Our meta-regression analysis, involving 171 response shift studies indicates that, on average, one out of five longitudinal PROM effects that are investigated for response shift result in the detection of response shift, when adjusting for sampling dependencies and controlling for sample- and effect-level variables. This result is consistent with our previous systematic review, based on 150 overlapping studies [8]. The results of our current analysis further indicate that two-fifths of the effect-level variance in response shift detection was explained by the variables we extracted from the included studies. Of those, the kind of response shift method accounted for almost half of this explained variance. Other notable variables influencing response shift detection included response shift type and the study quality control variables. Contrary to expectation, variation in response shift detection was not explained by population characteristics (sex, age, medical condition, and intervention).

The results highlight that variation in response shift detection is predominantly attributable to methodological differences across studies. Most importantly, the question arises as to why different response shift methods have a varying probability of detecting response shift, even after controlling for the other effect-level variables. Most model-based methods, such as latent variable methods based on Oort’s procedure [35], allow for multiple effects (e.g., effects for different response shift types and domains) that are tested simultaneously within the same model, thereby protecting against false positives. For instance, an overall test for response shift is inherently included in SEMs based on Oort’s procedure, which can help to prevent false positives even when multiple testing corrections are not performed after this overall test. Conversely, in design-based methods, each response shift effect (e.g., effects for different domains) is typically tested separately, in which case multiple testing could result in false positives (unless a method to control the familywise error rate, such as the Bonferroni method is used). This difference may, in part, explain why the probability of detecting response shift is lower for latent-variable methods than for the then-test. Of course, there is also a trade-off between controlling familywise error and statistical power to detect response shift effects; it might be that latent variable method studies are generally underpowered as compared to studies that use design-based methods. This argument is deemed less plausible as application of latent variable methods usually requires larger sample sizes (with more power to detect effects). Another possible explanation is that model-based methods have been more commonly applied in secondary data analyses, leading to a relatively smaller probability of detecting response shift. Conversely, the then-test can only be used in studies where it was included in the original design (i.e., there is no room for secondary analyses as response shift detection is most likely the focus of the primary analyses). Still, these arguments do not explain why the probability of detecting response shift is larger for regression methods and other methods. Therefore, we echo previous recommendations that response shift studies should evaluate and report on sample size requirements for the chosen statistical analyses [36, 37] (e.g., see Verdam [38] for a tutorial on power calculations for response shift investigations with SEM).

The magnitude of response shift was only available for effects investigated with then-test and SEM, where one-quarter of the variability of response shift effect sizes across samples was explained by population characteristics (sample-level variables) and only a tenth of the variability across response shift effects was explained by effect-level variables (including study design, the PROM, response shift method (then-test vs. SEM) and study quality control variables). However, effect sizes could be determined for only 132 (3.2%) of the 4176 effects investigated with SEM, whereas for effects investigated with then-test effect-size information was available for 90.0% of the investigated effects (i.e. 637 out of 708). Consequently, the effect size results predominantly represent studies using the then-test method and may not be representative of studies based on SEM or other methods for examining response shift. This points to a limitation of the response shift literature, as the preponderance of response shift studies do not report information about the magnitudes of effects and some methods do not enable effect size computation.

The average effect size of the response shift effects based on the then-test or SEM methods (including only those for which an effect size could be determined) was 0.30 (95% CI: 0.26–0.34) and all marginal effect sizes were above 0.20 (with two exceptions), indicating that they were far from negligible and predominantly ranged from small to moderate in size (based on Cohen’s guidelines [34]). To contextualize, most effects in PROM research are also of a small to moderate magnitude, as discussed previously [8] and in some instances, small effect sizes might be clinically relevant [39]. The largest marginal effect size (0.45) was found for analyses of time-period > 12 months and samples of mostly children/adolescents had the smallest marginal effect size (0.10). It is important to consider that the differences in marginal effect sizes across all of the explanatory and control variables are relatively small and nearly all of the CIs are overlapping, precluding firm conclusions about their relative importance.

The current meta-regression analysis adds to our previous descriptive systematic review by disentangling the variability induced by differences in population characteristics, study design, PROMs, response shift methods, and study quality. The results allow for direct comparisons of effects across different methods and other characteristics. A more specific strength is our extensive analysis of dependencies, which has guarded us against finding spurious effects. Finally, the current meta-regression analysis on response shift effects and effect sizes of quantitative response shift studies is the most comprehensive to date, including all investigated populations and methods.

Several limitations that were listed in our previously published descriptive systematic review [8] apply equally to the current meta-regression analysis, including: the omission of studies that are not reported in English; inclusion of studies that adopted different operationalizations of response shift and/or had incomplete reporting of study results and/or methodology; consideration of all detected effects as response shift effects, although their substantiation may be questioned; and inclusion of a limited number of explanatory variables that in the current meta-regression analysis explained less than half of the calculated variances. Another limitation is the lack of a formal assessment of study quality, as a meaningful assessment of study quality is hindered by the heterogeneity of the included studies (see section on risk of bias). Although we have included four control variables as indicators of study quality in all the analyses, it is important to keep in mind that due to a lack of direct assessment of study quality its influence on response shift results remains unknown. Relatedly, the extent to which response shift results are affected by the psychometric quality of PROMs was not investigated. As information about the reliability and validity of PROMs is rarely consistently reported we did not consider it feasible to include this information in the current analysis; its influence might be more easily investigated using simulation studies. Finally, exploration of potential interaction effects between the explanatory/control variables was not performed. This exploration was deemed too complex due to the limited number of observations and the relatively large number of explanatory variables. Nevertheless, insight into the dynamics between the different explanatory variables might be clinically meaningful and thus a relevant topic for future research.

The current meta-regression analysis, combined with our previous descriptive systematic review [8], provides insight into the variability of response shift results, i.e., how the detection of response shift and response shift effect sizes vary across populations, study designs, PROMs, response shift methods, and study quality criteria. Rather than focusing only on overall response shift effects, future research should aim to identify and understand the conditions under which response shift is more or less likely to occur. This may include person- versus variable-centered quantitative methods [40, 41], qualitative research, and examination of theoretical and philosophical perspectives [42]. The marginal probabilities and effect sizes of response shift effects may also be taken into account when interpreting the results of other comparable studies using PROMs by considering the potential occurrence and impact of response shift effects. Additionally, future studies on response shift detection need to be informed by the current insights into response shift effects and effect sizes, which may help with designing the study and interpreting the results [13]. Well-designed studies and contextualized interpretation of results are needed to improve our understanding of response shift.

## Electronic supplementary material

Below is the link to the electronic supplementary material.

Supplementary Material 1

## Data Availability

Data files are available at: Sawatzky, Richard; Mathilde Verdam; Yseulys Dubuy; Véronique Sébille, 2024, “Replication Data for Response Shift Results of Quantitative Research Using Patient-Reported Outcome Measures”, 10.5683/SP3/CLKZBL, Borealis.

## Code availability

Syntax for the various analyses are available at: Sawatzky, Richard; Mathilde Verdam; Yseulys Dubuy; Véronique Sébille, 2024, “Replication Data for Response Shift Results of Quantitative Research Using Patient-Reported Outcome Measures”, 10.5683/SP3/CLKZBL, Borealis.
