# Supplementary material for: Response shift results of quantitative research using patient-reported outcome measures: a meta-regression analysis
Source: Qual Life Res. 2024 Dec 9;34(5):1393–406. doi: 10.1007/s11136-024-03867-x (PMC12064579; doi:10.1007/s11136-024-03867-x)
Supplement: Supplementary file 1 — Supplementary Material 1 [file 11136_2024_3867_MOESM1_ESM.docx]

**Supplementary Material belonging to:** **Response shift results of quantitative research using patient-reported outcome measures: A meta-regression analysis**

Richard Sawatzky, Mathilde G. E. Verdam, Yseulys Dubuy, Tolulope T. Sajobi, Lara Russell, Oluwagbohunmi A. Awosoga, Ayoola Ademola, Jan R. Böhnke, Oluwaseyi Lawal, Anita Brobbey , Amelie Anota, Lisa M. Lix, Mirjam A. G. Sprangers, Véronique Sebille^6^, and the Response Shift – in Sync Working Group

Content:

**Table S1:** Model comparison results of the purposeful selection procedure for explaining variability in detection of response shift effects

**Table S2:** Parameter estimates of the final 2-level multivariate logistic regression model explaining variability in detection of response shift effects

**Table S3:** Model comparison results of the purposeful selection procedure for explaining variability in response shift effect sizes

**Table S4:** Parameter estimates of the final multivariate 3-level meta-regression model explaining variability in response shift effect sizes

**Appendix:** Reference list of included studies

**Table S1:** Model comparison results of the purposeful selection procedure for explaining variability in detection of response shift effects

|  | **Step 1** | |  | **Step 2** |  | **Step 3** |  |
| --- | --- | --- | --- | --- | --- | --- | --- |
| **Explanatory/control variables** | **LL Diff**^a^ **(DF**^b^**)** | | **p-value** | **LL Diff (DF)** | **p-value** | **LL Diff (DF)** | **p-value** |
| **Sample-level variables** |  | |  |  |  |  |  |
| **Population characteristics** |  | |  |  |  |  |  |
| **Sex** | 1.75 (3) | | 0.625 | n/a |  | 1.42 (3) | 0.700 |
| **Age** | 2.21 (3) | | 0.625 | n/a |  | 0.66 (3) | 0.882 |
| **Medical condition** | 27.31 (5) | | <.001 | 2.93 (5) | 0.711 | n/a |  |
| **Intervention** | 17.54 (3) | | 0.001 | 6.18 (3) | 0.103 | n/a |  |
| **Effect-level variables** |  | |  |  |  |  |  |
| **Study design characteristics** |  | |  |  |  |  |  |
| **Design** | 9.61 (1) | | 0.002 | 5.38 (1) | 0.020 | n/a |  |
| **Sample sizes** | 27.56 (4) | | <.001 | 13.51 (3) | 0.004 | n/a |  |
| **Time period classification** | 14.47 (4) | | 0.006 | 10.93 (4) | 0.027 | n/a |  |
| **PROM characteristics** |  | |  |  |  |  |  |
| **PROM types** | 7.89 (2) | | 0.019 | 4.34 (2) | 0.114 | n/a |  |
| **PROM domains** | 35.13 (5) | | <.001 | 26.86 (5) | <.001 | n/a |  |
| **Response shift type and methods** | |  |  |  |  |  |  |
| **Response shift type** | 190.03 (1) | | <.001 | 127.24 (1) | <.001 | n/a |  |
| **Response shift method** | 195.42 (3) | | <.001 | 85. 83 (3) | <.001 | n/a |  |

*Notes*: Each explanatory/control variable has at least two categories (i.e. one dummy variable for each category, except for the reference category; see Table 1 in the manuscript). When a variable has multiple categories, all associated dummy variables are tested simultaneously. ^a^ the difference in loglikelihoods between two models with and without the associated explanatory/control variable. ^b^ degrees of freedom. In Step 1 each explanatory/control variable is tested using a univariate model and a model without any explanatory/predictor variables. In Steps 2 and 3 each explanatory/control variable is tested using a multivariable model with and without the associated predictor/control variable. n/a indicates that the associated explanatory/control variable was not evaluated in that step of the procedure.

**Table S2:** Parameter estimates of the final 2-level multivariate logistic regression model explaining variability in detection of response shift effects

| **Explanatory/control variables** | **Estimated parameter** | | **Standard error** | **P-value** |
| --- | --- | --- | --- | --- |
| **Sample-level variables** |  | |  |  |
| **Population characteristics** |  | |  |  |
| **Sex** |  |  | |  |
| Mixed (ref) | n/a |  | |  |
| Only female | n/a |  | |  |
| Only male | n/a |  | |  |
| Other/Unknown | n/a |  | |  |
| **Age** |  | |  |  |
| Mostly adults (ref) | n/a | |  |  |
| Mostly Older Adults | n/a | |  |  |
| Mostly Children/Adolescents | n/a | |  |  |
| Other/Unknown | n/a | |  |  |
| **Medical condition** |  | |  |  |
| Yes: Cancer (ref) |  | |  |  |
| Yes: Orthopedic | -0.225 | | 0.639 | 0.724 |
| Yes: Stroke | -0.717 | | 0.631 | 0.256 |
| Yes: Mental Health | -0.317 | | 0.584 | 0.587 |
| Yes: Other | -0.313 | | 0.330 | 0.343 |
| No | -0.549 | | 0.633 | 0.386 |
| **Intervention** |  | |  |  |
| No/Unclear (ref) |  | |  |  |
| Yes: Medical | 0.555 | | 0.348 | 0.111 |
| Yes: Psychological | 0.442 | | 0.518 | 0.394 |
| Yes: Other/Unspecified | 1.003 | | 0.647 | 0.121 |
| **Effect-level variables** |  | |  |  |
| **Study design characteristics** |  | |  |  |
| **Design** |  | |  |  |
| Observational (ref) |  | |  |  |
| Experimental | -0.591 | | 0.368 | 0.108 |
| **Sample sizes** |  | |  |  |
| Q1 (< 57) | -0.886 | | 0.454 | 0.051 |
| Q2 (57 - 254) | 0.133 | | 0.353 | 0.707 |
| Q3 (255 - 410) | 0.511 | | 0.369 | 0.166 |
| Q4 (>411) (ref) |  | |  |  |
| **Time period classification** |  | |  |  |
| < 1 month | -0.405 | | 0.225 | 0.072 |
| 1-6 months (ref) |  | |  |  |
| >6 months - 12 months | 0.543 | | 0.254 | 0.032 |
| > 12 months | 0.715 | | 0.384 | 0.062 |
| Not reported | -0.557 | | 0.390 | 0.153 |
| **PROM characteristics** |  | |  |  |
| **PROM types** |  | |  |  |
| Generic PROMs (ref) |  | |  |  |
| Disease-Specific PROMs | -0.238 | | 0.245 | 0.330 |
| Individualized/other PROMs | 0.246 | | 0.256 | 0.337 |
| **PROM domains** |  | |  |  |
| General health/QOL (ref) |  | |  |  |
| Physical | -0.403 | | 0.182 | 0.027 |
| Psychological | -0.680 | | 0.195 | <.001 |
| Social | -0.565 | | 0.226 | 0.012 |
| Pain | 0.146 | | 0.245 | 0.522 |
| Other | -0.985 | | 0.305 | 0.001 |
| **Response shift type and method** |  | |  |  |
| **Response shift type** |  | |  |  |
| Recalibration (ref) |  | |  |  |
| Reprioritization/reconceptualization | -1.397 | | 0.127 | <.001 |
| **Response shift method^a^** |  | |  |  |
| Then-Test (ref) |  | |  |  |
| Latent variable method^b^ | -1.438 | | 0.256 | <.001 |
| Regression method^c^ | 1.305 | | 0.390 | <.001 |
| Other^d^ | 0.939 | | 0.338 | 0.005 |
| **Study quality control variables** |  | |  |  |
| **Primary data analysis** |  | |  |  |
| No (ref) |  | |  |  |
| Yes | 0.953 | | 0.308 | 0.002 |
| **Hypothesis about response shift** |  | |  |  |
| No (ref) |  | |  |  |
| Yes | -0.570 | | 0.226 | 0.012 |
| **Explanation of response shift** |  | |  |  |
| No (ref) |  | |  |  |
| Yes | 0.621 | | 0.215 | 0.004 |
| **Missing data reported** |  | |  |  |
| No (ref) |  | |  |  |
| Yes | 0.661 | | 0.339 | 0.051 |
| **Intercept** | -0.615 | | 0.531 | 0.247 |

*Notes*: ref = reference category of dummy-coded variables for which no direct estimates are available. n/a indicates that the associated explanatory/control variable was not included in the final model. ^a)^ See Table 1 of Sawatzky et al., 2023 [8] for detailed descriptions. ^b)^ Includes structural equation modeling, item response theory and Rasch measurement theory methods. ^c)^Includes regression methods with and without classification. ^d)^ Includes design-based methods other than the then-test (individualized methods, ideal scale approach, appraisal, change in importance ratings) as well as study-specific methods.

**Table S3:** Model comparison results of the purposeful selection procedure for explaining variability in response shift effect sizes

|  | **Step 1** | |  | | **Step 2** | |  | | **Step 3** | |  | |  |
| --- | --- | --- | --- | --- | --- | --- | --- | --- | --- | --- | --- | --- | --- |
| **Explanatory/control variables** | **LL Diff** ^a^ **(DF** ^b^**)** | | | **p-value** | | **LL Diff (DF)** | | **p-value** | | **LL Diff (DF)** | | **p-value** | |
| **Sample-level variables** |  | |  | |  | |  | |  | |  | |  |
| **Population characteristics** |  | |  | |  | |  | |  | |  | |  |
| **Sex** | 1.59 (3) | | 0.661 | | n/a | |  | | 1.10 (3) | | 0.775 | |  |
| **Age** | 8.39 (3) | | 0.039 | | 6.86 (3) | | 0.076 | | n/a | |  | |  |
| **Medical condition** | 15.48 (5) | | 0.009 | | 9.56 (5) | | 0.089 | | n/a | |  | |  |
| **Intervention** | 7.64 (3) | | 0.054 | | 3.16 (3) | | 0.368 | | n/a | |  | |  |
| **Effect-level variables** |  | |  | |  | |  | |  | |  | |  |
| **Study design characteristics** |  | |  | |  | |  | |  | |  | |  |
| **Design** | 4.13 (1) | | 0.042 | | 0.74 (1) | | 0.390 | | n/a | |  | |  |
| **Sample sizes** | 3.80 (3) | | 0.284 | | n/a | |  | | 3.00 (3) | | 0.391 | |  |
| **Time period classification** | 10.18 (4) | | 0.038 | | 12.42 (4) | | 0.014 | | n/a | |  | |  |
| **PROM characteristics** |  | |  | |  | |  | |  | |  | |  |
| **PROM types** | 14.23 (2) | | 0.001 | | 12.13 (2) | | 0.002 | | n/a | |  | |  |
| **PROM domains** | 26.16 (5) | | <.001 | | 24.72 (5) | | <.001 | | n/a | |  | |  |
| **Response shift type and methods** | |  |  | |  | |  | |  | |  | |  |
| **Response shift type** | 5.04 (1) | | 0.025 | | 3.59 (1) | | 0.058 | | n/a | |  | |  |
| **Response shift method** | 0.5 (1) | | 0.702 | | n/a | |  | | 0.88 (1) | | 0.347 | |  |

*Notes*: ^a^ difference in loglikelihoods between two models with and without the associated explanatory/control variable. ^b^ degrees of freedom. In Step 1 each explanatory/control variable is tested using a univariate model and a model without any explanatory/predictor variables. In Steps 2 and 3 each explanatory/control variable is tested using a multivariable model with and without the associated predictor/control variable. n/a indicates that the associated explanatory/control variable was not evaluated in that step of the procedure.

**Table S4:** Parameter estimates of the final multivariable 3-level meta-regression model explaining variability in response shift effect sizes

| **Explanatory/control variables** | **Estimated parameter** | **Standard error** | **P-value** |
| --- | --- | --- | --- |
| **Sample-level variance** | 0.021 | 0.005 | <.001 |
| **Population characteristics** |  |  |  |
| **Sex** |  |  |  |
| Mixed (ref) | n/a |  |  |
| Only female | n/a |  |  |
| Only male | n/a |  |  |
| Other/Unknown | n/a |  |  |
| **Age** |  |  |  |
| Mostly Adults (ref) |  |  |  |
| Mostly Older Adults | -0.014 | 0.047 | 0.767 |
| Mostly Children/Adolescents | -0.214 | 0.083 | 0.010 |
| Other/Unknown | -0.012 | 0.071 | 0.863 |
| **Medical condition** |  |  |  |
| Yes: Cancer (ref) |  |  |  |
| Yes: Orthopedic | 0.062 | 0.068 | 0.360 |
| Yes: Stroke | -0.153 | 0.096 | 0.110 |
| Yes: Mental Health | 0.119 | 0.114 | 0.297 |
| Yes: Other | -0.046 | 0.044 | 0.296 |
| No | 0.110 | 0.085 | 0.199 |
| **Intervention** |  |  |  |
| No/Unclear (ref) |  |  |  |
| Yes: Medical | 0.017 | 0.047 | 0.720 |
| Yes: Psychological | 0.118 | 0.071 | 0.098 |
| Yes: Other/Unspecified | 0.053 | 0.089 | 0.551 |
| **Effect-level variance** | 0.017 | 0.001 | <.001 |
| **Study design characteristics** |  |  |  |
| **Design** |  |  |  |
| Observational (ref) |  |  |  |
| Experimental | 0.452 | 0.051 | 0.405 |
| **Sample size** |  |  |  |
| Q1 (< 57) | n/a |  |  |
| Q2 (57 - 254) | n/a |  |  |
| Q3 (255 - 410) | n/a |  |  |
| Q4 (>411) (ref) | n/a |  |  |
| Not reported | n/a |  |  |
| **Time period classification** |  |  |  |
| < 1 month | -0.019 | 0.022 | 0.395 |
| 1-6 months (ref) |  |  |  |
| >6 months - 12 months | 0.015 | 0.040 | 0.706 |
| > 12 months | 0.154 | 0.052 | 0.003 |
| Not reported | -0.045 | 0.035 | 0.205 |
| **PROM characteristics** |  |  |  |
| **PROM type** |  |  |  |
| Generic PROMs (ref) |  |  |  |
| Disease-Specific PROMs | 0.101 | 0.031 | 0.001 |
| Individualized/other PROM | 0.012 | 0.036 | 0.736 |
| **PROM domain** |  |  |  |
| General health/QOL (ref) |  |  |  |
| Physical | -0.026 | 0.023 | 0.265 |
| Psychological | -0.038 | 0.025 | 0.127 |
| Social | 0.010 | 0.029 | 0.726 |
| Pain | 0.077 | 0.030 | 0.011 |
| Other | -0.004 | 0.031 | 0.900 |
| **Response shift type and method** |  |  |  |
| **Response shift type** |  |  |  |
| Recalibration (ref) |  |  |  |
| Reprioritization/reconceptualization | -0.085 | 0.038 | 0.025 |
| **Response shift method** |  |  |  |
| Then-Test (ref) |  |  |  |
| Structural equation modeling | 0.050 | 0.039 | 0.204 |
| **Study quality control variables** |  |  |  |
| **Primary data analysis** |  |  |  |
| No (ref) |  |  |  |
| Yes | 0.038 | 0.046 | 0.405 |
| **Hypothesis about response shift** |  |  |  |
| No (ref) |  |  |  |
| Yes | 0.024 | 0.031 | 0.435 |
| **Explanation of response shift** |  |  |  |
| No (ref) |  |  |  |
| Yes | 0.036 | 0.022 | 0.109 |
| **Missing data reported** |  |  |  |
| No (ref) |  |  |  |
| Yes | -0.003 | 0.049 | 0.944 |
| **Intercept** | 0.168 | 0.068 | 0.013 |

*Notes*: ref = reference category of dummy-coded variables for which no direct estimates are available. n/a indicates that the associated explanatory/control variable was not included in the final model.

**Appendix: Reference list of included studies**

1. Abolhassani, N., Santos-Eggimann, B., Bula, C., Goy, R., Guessous, I., & Henchoz, Y. (2019). Temporal changes in importance of quality of life domains: a longitudinal study in community-dwelling Swiss older people. Quality of Life Research, 28(2), 421-428. https://doi.org/10.1007/s11136-018-1983-4

2. Aburub, A. S., Gagnon, B., Ahmed, S., Rodríguez, A. M., & Mayo, N. E. (2018). Impact of reconceptualization response shift on rating of quality of life over time among people with advanced cancer. Supportive Care in Cancer, 26(9), 3063-3071. https://doi.org/10.1007/s00520-018-4156-7

3. Addington-Hall, J., Hunt, K., Rowsell, A., Heal, R., Hansford, P., Monroe, B., & Sykes, N. (2014). Development and initial validation of a new outcome measure for hospice and palliative care: the St Christopher's Index of Patient Priorities (SKIPP). BMJ Supportive & Palliative Care, 4(2), 175-181. https://doi.org/bmjspcare-2012-000352

4. Ahmed, S., Bourbeau, J., Maltais, F., & Mansour, A. (2009). The Oort structural equation modeling approach detected a response shift after a COPD self-management program not detected by the Schmitt technique. Journal of Clinical Epidemiology, 62(11), 1165-1172. https://doi.org/S0895-4356(09)00122-X

5. Ahmed, S., Mayo, N., Scott, S., Kuspinar, A., & Schwartz, C. (2011). Using latent trajectory analysis of residuals to detect response shift in general health among patients with multiple sclerosis article. Quality of Life Research, 20(10), 1555-1560. https://doi.org/10.1007/s11136-011-0005-6

6. Ahmed, S., Mayo Nancy, E., Wood-Dauphinee, S., Hanley James, A., & Cohen, S. R. (2005). The structural equation modeling technique did not show a response shift, contrary to the results of the then test and the individualized approaches. Journal of clinical epidemiology, 58(11), 1125-1133.

7. Ahmed, S., Mayo, N. E., Corbiere, M., Wood-Dauphinee, S., Hanley, J., & Cohen, R. (2005). Change in quality of life of people with stroke over time: true change or response shift? Quality of Life Research, 14(3), 611-627. https://doi.org/10.1007/s11136-004-3708-0

8. Ahmed, S., Mayo, N. E., Wood-Dauphinee, S., Hanley, J. A., & Cohen, S. R. (2004). Response shift influenced estimates of change in health-related quality of life poststroke. Journal of Clinical Epidemiology, 57(6), 561-570. https://doi.org/S0895435603004189

9. Ahmed, S., Mayo, N. E., Wood-Dauphinee, S., Hanley, J. A., & Cohen, S. R. (2005). Using the Patient Generated Index to evaluate response shift post-stroke. Quality of Life Research, 14(10), 2247-2257. https://doi.org/10.1007/s11136-005-8118-4

10. Ahmed, S., Sawatzky, R., Levesque, J. F., Ehrmann-Feldman, D., & Schwartz, C. E. (2014). Minimal evidence of response shift in the absence of a catalyst. Quality of Life Research, 23(9), 2421-2430. https://doi.org/10.1007/s11136-014-0699-3

11. Andrykowski, M. A., Donovan, K. A., & Jacobsen, P. B. (2009). Magnitude and correlates of response shift in fatigue ratings in women undergoing adjuvant therapy for breast cancer. Journal of Pain and Symptom Management, 37(3), 341-351. https://doi.org/10.1016/j.jpainsymman.2008.03.015

12. Anota, A., Bascoul-Mollevi, C., Conroy, T., Guillemin, F., Velten, M., Jolly, D., Mercier, M., Causeret, S., Cuisenier, J., Graesslin, O., Hamidou, Z., & Bonnetain, F. (2014). Item response theory and factor analysis as a mean to characterize occurrence of response shift in a longitudinal quality of life study in breast cancer patients. Health and Quality of Life Outcomes, 12(1), 32. https://doi.org/10.1186/1477-7525-12-32

13. Arthur, J., Watts, T., Davies, R., Manchaiah, V., & Slater, J. (2016). An exploratory study identifying a possible response shift phenomena of the Glasgow Hearing Aid Benefit Profile. Audiology Research, 6(2), 44-48. https://doi.org/10.4081/audiores.2016.152

14. Auneau-Enjalbert, L., Blanchin, M., Giral, M., Meurette, A., Morelon, E., Albano, L., Hardouin, J. B., & Sebille, V. (2022). Investigation of measurement invariance in longitudinal health-related quality of life in preemptive or previously dialyzed kidney transplant recipients. Quality of Life Research, 31(2), 607-620. https://doi.org/10.1007/s11136-021-02916-z

15. Balain, B., Ennis, O., Kanes, G., Singhal, R., Roberts, S. N., Rees, D., & Kuiper, J. H. (2009). Response shift in self-reported functional scores after knee microfracture for full thickness cartilage lesions. Osteoarthritis Cartilage, 17(8), 1009-1013. https://doi.org/S1063-4584(09)00046-6

16. Bar-On, D., Lazar, A., & Amir, M. (2000). Quantitative assessment of response shift in QOL research. Social Indicators Research, 49(1), 37-49. https://doi.org/10.1023/a:1006933612340

17. Barclay, R., & Tate, R. B. (2014). Response shift recalibration and reprioritization in health-related quality of life was identified prospectively in older men with and without stroke. Journal of Clinical Epidemiology, 67(5), 500-507. https://doi.org/10.1016/j.jclinepi.2013.12.003

18. Barclay-Goddard, R., Lix, L. M., Tate, R., Weinberg, L., & Mayo, N. E. (2009). Response shift was identified over multiple occasions with a structural equation modeling framework. Journal of Clinical Epidemiology, 62(11), 1181-1188. https://doi.org/10.1016/j.jclinepi.2009.03.014

19. Barclay-Goddard, R., Lix, L. M., Tate, R., Weinberg, L., & Mayo, N. E. (2011). Health-related quality of life after stroke: Does response shift occur in self-perceived physical function? Archives of Physical Medicine and Rehabilitation, 92(11), 1762-1769. https://doi.org/10.1016/j.apmr.2011.06.013

20. Bastianelli, A., Gius, E., & Cipolletta, S. (2016). Changes over time in the quality of life, prolonged grief and family strain of family caregivers of patients in vegetative state: A pilot study. Journal of Health Psychol, 21(5), 844-852. https://doi.org/1359105314539533

21. Bernhard, J., Hürny, C., Maibach, R., Herrmann, R., & Laffer, U. (1999). Quality of life as subjective experience: reframing of perception in patients with colon cancer undergoing radical resection with or without adjuvant chemotherapy. Annals of Oncology, 10(7), 775-782. https://doi.org/10.1023/a:1008311918967

22. Bernhard, J., Lowy, A., Maibach, R., & Hurny, C. (2001). Response shift in the perception of health for utility evaluation: an explorative investigation. European Journal Cancer, 37(14), 1729-1735. https://doi.org/S0959-8049(01)00196-4

23. Bernhard, J., Lowy, A., Mathys, N., Herrmann, R., & Hurny, C. (2004). Health related quality of life: a changing construct? Quality of Life Research, 13(7), 1187-1197. https://doi.org/10.1023/B:QURE.0000037485.59681.7d

24. Berry, D. L., Pett, M. A., Clayton, M. F., Beck, S. L., & Tavernier, S. S. (2011). Validity of the Patient Generated Index as a quality-of-life measure in radiation oncology. Oncology Nursing Forum, 38(3), 319-329. https://doi.org/10.1188/11.Onf.319-329

25. Blanchin, M., Sébille, V., Guilleux, A., & Hardouin, J.-B. (2016). The Guttman errors as a tool for response shift detection at subgroup and item levels. Quality of Life Research, 25(6), 1385-1393. https://doi.org/10.1007/s11136-016-1268-8

26. Boucekine, M., Boyer, L., Baumstarck, K., Millier, A., Ghattas, B., Auquier, P., & Toumi, M. (2015). Exploring the response shift effect on the quality of life of patients with schizophrenia: an application of the random forest method. Medical Decision Making, 35(3), 388-397. https://doi.org/0272989X14559273

27. Boucekine, M., Loundou, A., Baumstarck, K., Minaya-Flores, P., Pelletier, J., Ghattas, B., & Auquier, P. (2013). Using the random forest method to detect a response shift in the quality of life of multiple sclerosis patients: a cohort study. BMC Medical Research Methodology, 13, 20. https://doi.org/1471-2288-13-20

28. Brill, I. T., Stark, T., Wigers, L., & Brill, S. M. (2023). Response shift in hearing related quality of life after cochlear implantation – effect size and clinical significance: a then-test study. Health and Quality of Life Outcomes, 21(1), 1-10. https://doi.org/10.1186/s12955-023-02118-w

29. Brinksma, A., Tissing, W. J., Sulkers, E., Kamps, W. A., Roodbol, P. F., & Sanderman, R. (2014). Exploring the response shift phenomenon in childhood patients with cancer and its effect on health-related quality of life. Oncology Nursing Forum, 41(1), 48-56. https://doi.org/J7H880604025V7J1

30. Broberger, E., Sprangers, M., & Tishelman, C. (2006). Do internal standards of quality of life change in lung cancer patients? Nursing Research, 55(4), 274-282.

31. Brook, J., Akin, B. A., Lloyd, M., Bhattarai, J., & McDonald, T. P. (2016). The use of prospective versus retrospective pretests with child-welfare involved families. Journal of Child and Family Studies, 25(9), 2740-2752. https://doi.org/10.1007/s10826-016-0446-1

32. Bulteau, S., Blanchin, M., Pere, M., Poulet, E., Brunelin, J., Sauvaget, A., & Sebille, V. (2023). Impact of response shift effects in the assessment of self-reported depression during treatment: Insights from a rTMS versus Venlafaxine randomized controlled trial. Journal of Psychiatric Research, 160, 117-125. https://doi.org/S0022-3956(23)00075-4

33. Chen, H., Zhu, L., Zhou, R., Liu, P., Lu, X., Patrick, D. L., Edwards, T. C., & Wang, H. (2021). Detecting response shift in health-related quality of life measurement among patients with hypertension using structural equation modeling. Health and Quality of Life Outcomes, 19(1). https://doi.org/10.1186/s12955-021-01732-w

34. Chen, P.-Y., Jan, Y.-W., & Yang, C.-M. (2017). Are the Insomnia Severity Index and Pittsburgh Sleep Quality Index valid outcome measures for Cognitive Behavioral Therapy for Insomnia? Inquiry from the perspective of response shifts and longitudinal measurement invariance in their Chinese versions. Sleep Medicine, 35, 35-40. https://doi.org/10.1016/j.sleep.2017.04.003

35. Chen, P. Y., & Yang, C. M. (2020). Consequences of ignoring the response-shift and measure non-invariant items in sleep studies: an empirical data based simulation of the treatment effect of CBT-I on dysfunctional sleep beliefs. Sleep Medicine, 74, 99-108. https://doi.org/S1389-9457(20)30212-4

36. Chin, K., Fukuhara, S., Takahashi, K., Sumi, K., Matsumoto, H., Niimi, A., Hattori, N., Mishima, M., & Nakamura, T. (2004). Response shift in perception of sleepiness in obstructive sleep apnea-hypopnea syndrome before and after treatment with nasal CPAP. Sleep, 27(3), 490-493.

37. Chow, E., Chiu, H., Doyle, M., Hruby, G., Holden, L., Barnes, E. A., Tsao, M., Mallia, G., Harris, K., & Danjoux, C. (2007). Patient expectation of the partial response and response shift in pain score. Support Cancer Therapy, 4(2), 110-118. https://doi.org/L2317620361N2733

38. Dabakuyo, T. S., Guillemin, F., Conroy, T., Velten, M., Jolly, D., Mercier, M., Causeret, S., Cuisenier, J., Graesslin, O., Gauthier, M., & Bonnetain, F. (2012). Response shift effects on measuring post-operative quality of life among breast cancer patients: a multicenter cohort study. Quality of Life Research, 22(1), 1-11. https://doi.org/10.1007/s11136-012-0135-5

39. de Beurs, D. P., Fokkema, M., de Groot, M. H., de Keijser, J., & Kerkhof, A. J. (2015). Longitudinal measurement invariance of the Beck Scale for Suicide Ideation. Psychiatry Res, 225(3), 368-373. https://doi.org/S0165-1781(14)00999-8

40. DeConde, A. S., Bodner, T. E., Mace, J. C., & Smith, T. L. (2014). Response Shift in Quality of Life After Endoscopic Sinus Surgery for Chronic Rhinosinusitis. JAMA Otolaryngology–Head & Neck Surgery, 140(8), 712-719. https://doi.org/10.1001/jamaoto.2014.1045

41. Dempster, M., Carney, R., & McClements, R. (2010). Response shift in the assessment of quality of life among people attending cardiac rehabilitation. British Journal of Health Psychology, 15(2), 307-319. https://doi.org/10.1348/135910709x464443

42. Dubuy, Y., Sébille, V., Grall-Bronnec, M., Challet-Bouju, G., Blanchin, M., & Hardouin, J.-B. (2021). Evaluation of the link between the Guttman errors and response shift at the individual level. Quality of Life Research, 31(1), 61-73. https://doi.org/10.1007/s11136-021-03015-9

43. Echteld, M. A., van Zuylen, L., Bannink, M., Witkamp, E., & Van der Rijt, C. C. (2007). Changes in and correlates of individual quality of life in advanced cancer patients admitted to an academic unit for palliative care. Palliative Medicine, 21(3), 199-205. https://doi.org/21/3/199

44. Echteld Michael, A., Deliens, L., Ooms Marcel, E., Ribbe Miel, W., van der, W., & Gerrit. (2005). Quality of life change and response shift in patients admitted to palliative care units: a pilot study. Palliative Medicine, 19(5), 381-388.

45. Edelaar-Peeters, Y., & Stiggelbout, A. M. (2013). Anticipated adaptation or scale recalibration? Health and Quality of Life Outcomes, 11, 171. https://doi.org/1477-7525-11-171

46. Felix, J., Becker, C., Vogl, M., Buschner, P., Plotz, W., & Leidl, R. (2019). Patient characteristics and valuation changes impact quality of life and satisfaction in total knee arthroplasty - results from a German prospective cohort study. Health and Quality of Life Outcomes, 17(1), 180. https://doi.org/10.1186/s12955-019-1237-3

47. Finkelstein, J. A., Quaranto, B. R., & Schwartz, C. E. (2013). Threats to the internal validity of spinal surgery outcome assessment: recalibration response shift or implicit theories of change? Applied Research in Quality of Life, 9(2), 215-232. https://doi.org/10.1007/s11482-013-9221-2

48. Friedrich, M., Karoff, J., & Hinz, A. (2019). Response shift effects in patients’ assessments of their quality of life after cardiac rehabilitation. Quality of Life Research, 28(9), 2609-2620. https://doi.org/10.1007/s11136-019-02195-9

49. Friedrich, M., Zenger, M., & Hinz, A. (2019). Response shift effects of quality of life assessments in breast cancer survivors. European Journal Cancer Care, 28(2), e12979. https://doi.org/10.1111/ecc.12979

50. Gadermann, A. M., Sawatzky, R., Palepu, A., Hubley, A. M., Zumbo, B. D., Aubry, T., Farrell, S., & Hwang, S. W. (2017). Minimal impact of response shift for SF-12 mental and physical health status in homeless and vulnerably housed individuals: an item-level multi-group analysis. Quality of Life Research, 26(6), 1463-1472. https://doi.org/10.1007/s11136-016-1464-6

51. Galenkamp, H., Huisman, M., Braam, A. W., & Deeg, D. J. (2012). Estimates of prospective change in self-rated health in older people were biased owing to potential recalibration response shift. Journal of Clinical Epidemiology, 65(9), 978-988.

52. Gandhi, P. K., Ried, L. D., Huang, I. C., Kimberlin, C. L., & Kauf, T. L. (2012). Assessment of response shift using two structural equation modeling techniques. Quality of Life Research, 22(3), 461-471. https://doi.org/10.1007/s11136-012-0171-1

53. Gandhi, P. K., Schwartz, C. E., Reeve, B. B., DeWalt, D. A., Gross, H. E., & Huang, I. C. (2016). An item-level response shift study on the change of health state with the rating of asthma-specific quality of life: a report from the PROMIS® Pediatric Asthma Study. Quality of Life Research, 25(6), 1349-1359. https://doi.org/10.1007/s11136-016-1290-x

54. Gerlich, C., Schuler, M., Jelitte, M., Neuderth, S., Flentje, M., Graefen, M., Kruger, A., Mehnert, A., & Faller, H. (2016). Prostate cancer patients' quality of life assessments across the primary treatment trajectory: 'True' change or response shift? Acta Oncologica, 55(7), 814-820. https://doi.org/10.3109/0284186X.2015.1136749

55. Gillison, F., Skevington, S., & Standage, M. (2008). Exploring response shift in the quality of life of healthy adolescents over 1 year. Quality of Life Research, 17(7), 997-1008. https://doi.org/10.1007/s11136-008-9373-y

56. Guilleux, A., Blanchin, M., Vanier, A., Guillemin, F., Falissard, B., Schwartz, C. E., Hardouin, J. B., & Sebille, V. (2015). RespOnse Shift ALgorithm in Item response theory (ROSALI) for response shift detection with missing data in longitudinal patient-reported outcome studies. Quality of Life Research, 24(3), 553-564. https://doi.org/10.1007/s11136-014-0876-4

57. Haagsma, J. A., Spronk, I., de Jongh, M. A. C., Bonsel, G. J., & Polinder, S. (2020). Conventional and retrospective change in health-related quality of life of trauma patients: an explorative observational follow-up study. Health and Quality of Life Outcomes, 18(1), 1-13. https://doi.org/10.1186/s12955-020-01404-1

58. Hagedoorn, M., Sneeuw, K. C., & Aaronson, N. K. (2002). Changes in physical functioning and quality of life in patients with cancer: response shift and relative evaluation of one's condition. Journal of Clinical Epidemiology, 55(2), 176-183. https://doi.org/S0895435601004383

59. Hamidou, Z., Dabakuyo-Yonli, T. S., Guillemin, F., Conroy, T., Velten, M., Jolly, D., Causeret, S., Graesslin, O., Gauthier, M., Mercier, M., & Bonnetain, F. (2014). Impact of response shift on time to deterioration in quality of life scores in breast cancer patients. PLoS One, 9(5), e96848. https://doi.org/PONE-D-13-36716

60. Hammas, K., Sébille, V., Brisson, P., Hardouin, J.-B., & Blanchin, M. (2020). How to investigate the effects of groups on changes in longitudinal patient-reported outcomes and response shift using Rasch models. Frontiers in Psychology, 11. https://doi.org/10.3389/fpsyg.2020.613482

61. Hinz, A., Finck Barboza, C., Zenger, M., Singer, S., Schwalenberg, T., & Stolzenburg, J. U. (2011). Response shift in the assessment of anxiety, depression and perceived health in urologic cancer patients: an individual perspective. European Journal Cancer Care, 20(5), 601-609. https://doi.org/10.1111/j.1365-2354.2011.01256.x

62. Hinz, A., Karoff, J., Kittel, J., Brähler, E., Zenger, M., Schmalbach, B., & Kocalevent, R.-D. (2020). Associations between self-rated health and the assessments of anchoring vignettes in cardiovascular patients. International Journal of Clinical and Health Psychology, 20(2), 100-107. https://doi.org/10.1016/j.ijchp.2020.04.001

63. Hoch, J. M., Jamali, B. E., Hoch, M. C., & Powden, C. J. (2019). Response shift after a 4-week multimodal intervention for chronic ankle instability. Journal of Athletic Training, 54(4), 397-402. https://doi.org/10.4085/1062-6050-345-17

64. Höfer, S., Pfaffenberger, N., Renn, D., Platter, M., & Ring, L. (2010). Coronary intervention improves disease specific health-related quality of life but not individualised quality of life: a potential response shift effect? Applied Research in Quality of Life, 6(1), 81-90. https://doi.org/10.1007/s11482-010-9117-3

65. Hollman, F., Wessel, R. N., & Wolterbeek, N. (2016). Response shift of the Western Ontario Rotator Cuff index in patients undergoing arthroscopic rotator cuff repair. Journal of Shoulder and Elbow Surgery, 25(12), 2011-2018. https://doi.org/S1058-2746(16)30153-7

66. Hosseini, B., Nedjat, S., Zendehdel, K., Majdzadeh, R., Nourmohammadi, A., & Montazeri, A. (2017). Response shift in quality of life assessment among cancer patients: A study from Iran. Medical Journal of the Islamic Republic of Iran, 31(1), 798-803. https://doi.org/10.14196/mjiri.31.120

67. Howard, J. S., Mattacola, C. G., Mullineaux, D. R., English, R. A., & Lattermann, C. (2014). Influence of response shift on early patient-reported outcomes following autologous chondrocyte implantation. Knee Surg Sports Traumatol Arthrosc, 22(9), 2163-2171. https://doi.org/10.1007/s00167-013-2654-1

68. Huang, I. C., Sim, J. A., Srivastava, D., Krull, K. R., Ness, K. K., Robison, L. L., Baker, J. N., Hudson, M. M., & Schwartz, C. E. (2023). Response-shift effects in childhood cancer survivors: A prospective study. Psycho-Oncology, 32(7), 1085-1095. https://doi.org/10.1002/pon.6150

69. Inoue, M., Kasai, T., Kawana, F., & Narui, K. (2007). Response shift of subjective sleepiness in patients with obstructive sleep apnea-hypopnea syndrome. Sleep and Biological Rhythms, 5(2), 95-99. https://doi.org/10.1111/j.1479-8425.2007.00258.x

70. Ito, N., Ishiguro, M., Tanaka, M., Tokunaga, K., Sugihara, K., & Kazuma, K. (2010). Response shift in quality-of-life assessment in patients undergoing curative surgery with permanent colostomy: a preliminary study. Gastroenterol Nursing, 33(6), 408-412. https://doi.org/00001610-201011000-00004

71. Jabrayilov, R., Emons, W. H. M., de Jong, K., & Sijtsma, K. (2017). Longitudinal measurement invariance of the Dutch Outcome Questionnaire-45 in a clinical sample. Quality of Life Research, 26(6), 1473-1481. https://doi.org/10.1007/s11136-017-1500-1

72. Jakola, A. S., Solheim, O., Gulati, S., & Sagberg, L. M. (2016). Is there a response shift in generic health-related quality of life 6 months after glioma surgery? Acta Neurochirurgica, 159(2), 377-384. https://doi.org/10.1007/s00701-016-3040-9

73. Jansen, S. J., Stiggelbout, A. M., Nooij, M. A., Noordijk, E. M., & Kievit, J. (2000). Response shift in quality of life measurement in early-stage breast cancer patients undergoing radiotherapy. Quality of Life Research, 9(6), 603-615. https://doi.org/10.1023/a:1008928617014

74. Joore, M. A., Potjewijd, J., Timmerman, A. A., & Anteunis, L. J. (2002). Response shift in the measurement of quality of life in hearing impaired adults after hearing aid fitting. Quality of Life Research, 11(4), 299-307. https://doi.org/10.1023/a:1015598807510

75. Kievit, W., Hendrikx, J., Stalmeier, P. F., van de, L., M, A., Van, R., P, L., & Adang, E. M. (2010). The relationship between change in subjective outcome and change in disease: a potential paradox. Quality of Life Research, 19(7), 985-994.

76. Kimura, A., Arakawa, H., Noda, K., Yamazaki, S., Hara, E. S., Mino, T., Matsuka, Y., Mulligan, R., & Kuboki, T. (2012). Response shift in oral health-related quality of life measurement in patients with partial edentulism. Journal of Oral Rehabil, 39(1), 44-54. https://doi.org/10.1111/j.1365-2842.2011.02241.x

77. King-Kallimanis, B. L., Oort, F. J., & Garst, G. J. A. (2010). Using structural equation modelling to detect measurement bias and response shift in longitudinal data. AStA Advances in Statistical Analysis, 94(2), 139-156. https://doi.org/10.1007/s10182-010-0129-y

78. King-Kallimanis, B. L., Oort, F. J., Nolte, S., Schwartz, C. E., & Sprangers, M. A. (2011). Using structural equation modeling to detect response shift in performance and health-related quality of life scores of multiple sclerosis patients. Quality of Life Research, 20(10), 1527-1540. https://doi.org/9844

79. King-Kallimanis, B. L., Oort, F. J., Visser, M. R., & Sprangers, M. A. (2009). Structural equation modeling of health-related quality-of-life data illustrates the measurement and conceptual perspectives on response shift. Journal of Clinical Epidemiology, 62(11), 1157-1164. https://doi.org/S0895-4356(09)00114-0

80. Korfage Ida, J., de, K., Harry, J., & Essink-Bot, M.-L. (2007). Response shift due to diagnosis and primary treatment of localized prostate cancer: a then-test and a vignette study. Quality of Life Research, 16(10), 1627-1634.

81. Kubota, Y., Yoneda, K., Nakai, K., Katsuura, J., Moriue, T., Matsuoka, Y., Miyamoto, I., & Ohya, Y. (2009). Effect of sequential applications of topical tacrolimus and topical corticosteroids in the treatment of pediatric atopic dermatitis: an open-label pilot study. Journal of the American Academy of Dermatology, 60(2), 212-217. https://doi.org/S0190-9622(08)01215-2

82. Kuijer, R. G., De Ridder, D. T. D., Colland, V. T., Schreurs, K. M. G., & Sprangers, M. A. G. (2007). Effects of a short self-management intervention for patients with asthma and diabetes: Evaluating health-related quality of life using then-test methodology. Psychology & Health, 22(4), 387-411. https://doi.org/10.1080/14768320600843226

83. Kunz, S., Carrard, V., Galvis Aparicio, M., Scheel-Sailer, A., Fekete, C., Lude, P., Post, M. W. M., & Westphal, M. (2022). Longitudinal measurement invariance of the international spinal cord injury quality of life basic data set (SCI-QoL-BDS) during spinal cord injury/disorder inpatient rehabilitation. Quality of Life Research, 31(4), 1247-1256. https://doi.org/10.1007/s11136-021-03027-5

84. Kvam, A. K., Wisløff, F., & Fayers, P. M. (2010). Minimal important differences and response shift in health-related quality of life; a longitudinal study in patients with multiple myeloma. Health and Quality of Life Outcomes, 8(1), 79. https://doi.org/10.1186/1477-7525-8-79

85. Lawal, O. A., Awosoga, O., Santana, M. J., James, M. T., Wilton, S. B., Norris, C. M., Lix, L. M., & Sajobi, T. T. (2022). Measurement invariance of the Seattle Angina Questionnaire in coronary artery disease. Quality of Life Research, 31(4), 1223-1236. https://doi.org/10.1007/s11136-021-02987-y

86. Lepore, S. J., & Eton, D. T. (2000). Response shifts in prostate cancer patients: An evaluation of suppressor and buffer models. In Adaptation to changing health: Response shift in quality-of-life research. (pp. 37-51).

87. Li, Y., & Rapkin, B. (2009). Classification and regression tree uncovered hierarchy of psychosocial determinants underlying quality-of-life response shift in HIV/AIDS. Journal of Clinical Epidemiology, 62(11), 1138-1147. https://doi.org/S0895-4356(09)00113-9

88. Li, Y., & Schwartz, C. E. (2011). Data mining for response shift patterns in multiple sclerosis patients using recursive partitioning tree analysis. Quality of Life Research, 20(10), 1543-1553. https://doi.org/10.1007/s11136-011-0004-7

89. Liu, J. J., & Davis, G. E. (2015). The significance of response shift in sinus surgery outcomes. Int Forum Allergy Rhinol, 5(1), 55-59. https://doi.org/10.1002/alr.21420

90. Lix, L. M., Chan, E. K., Sawatzky, R., Sajobi, T. T., Liu, J., Hopman, W., & Mayo, N. (2016). Response shift and disease activity in inflammatory bowel disease. Quality of Life Research, 25(7), 1751-1760. https://doi.org/10.1007/s11136-015-1188-z

91. Lix, L. M., Sajobi, T. T., Sawatzky, R., Liu, J., Mayo, N. E., Huang, Y., Graff, L. A., Walker, J. R., Ediger, J., Clara, I., Sexton, K., Carr, R., & Bernstein, C. N. (2013). Relative importance measures for reprioritization response shift. Quality of Life Research, 22(4), 695-703. https://doi.org/10.1007/s11136-012-0198-3

92. Machuca, C., Vettore, M. V., Krasuska, M., Baker, S. R., & Robinson, P. G. (2017). Using classification and regression tree modelling to investigate response shift patterns in dentine hypersensitivity. BMC Medical Research Methodology, 17(1), 120. https://doi.org/10.1186/s12874-017-0396-3

93. Machuca, C., Vettore, M. V., & Robinson, P. G. (2020). How peoples’ ratings of dental implant treatment change over time? Quality of Life Research, 29(5), 1323-1334. https://doi.org/10.1007/s11136-019-02408-1

94. Mayo, N. E., Scott, S. C., & Ahmed, S. (2009). Case management poststroke did not induce response shift: the value of residuals. Journal of Clinical Epidemiology, 62(11), 1148-1156. https://doi.org/10.1016/j.jclinepi.2009.03.020

95. Mayo, N. E., Scott, S. C., Bernstein, C. N., & Lix, L. M. (2015). How are you? Do people with inflammatory bowel disease experience response shift on this question? Health and Quality of Life Outcomes, 13(1), 52. https://doi.org/10.1186/s12955-015-0232-6

96. Mayo, N. E., Scott, S. C., Dendukuri, N., Ahmed, S., & Wood-Dauphinee, S. (2008). Identifying response shift statistically at the individual level. Quality of Life Research, 17(4), 627-639. https://doi.org/10.1007/s11136-008-9329-2

97. McPhail, S., & Haines, T. (2010). Response shift, recall bias and their effect on measuring change in health-related quality of life amongst older hospital patients. Health and Quality of Life Outcomes, 8(1), 65-65. https://doi.org/10.1186/1477-7525-8-65

98. Modarresi, S., & Walton, D. M. (2021). Reliability, discriminative accuracy, and an exploration of response shift as measured using the satisfaction and Recovery Index over 12 months from musculoskeletal trauma. Musculoskeletal Science and Practice, 51, 102300. https://doi.org/10.1016/j.msksp.2020.102300

99. Mollerup, A., & Johansen, J. D. (2015). Response shift in severity assessment of hand eczema with visual analogue scales. Contact Dermatitis, 72(3), 178-183. https://doi.org/10.1111/cod.12335

100. Muller, F., Verdam, M. G. E., Oort, F. J., Riper, H., van Straten, A., Verdonck-de Leeuw, I. M., Sprangers, M. A. G., & Knoop, H. (2023). Response shift after cognitive behavioral therapy targeting severe fatigue: explorative analysis of three randomized controlled trials. International Journal of Behavioral Medicine, 30(4), 473-485. https://doi.org/10.1007/s12529-022-10111-8

101. Murata, T., Suzukamo, Y., Shiroiwa, T., Taira, N., Shimozuma, K., Ohashi, Y., & Mukai, H. (2020). Response shift-adjusted treatment effect on health-related quality of life in a randomized controlled trial of Taxane versus S-1 for metastatic breast cancer: structural equation modeling. Value in Health, 23(6), 768-774. https://doi.org/S1098-3015(20)30129-7

102. Murray, A. L., McKenzie, K., Murray, K., & Richelieu, M. (2018). Examining response shifts in the Clinical Outcomes in Routine Evaluation- Outcome Measure (CORE-OM). British Journal of Guidance & Counselling, 48(2), 276-288. https://doi.org/10.1080/03069885.2018.1483007

103. Nagl, M., & Farin, E. (2012). Response shift in quality of life assessment in patients with chronic back pain and chronic ischaemic heart disease. Disabil Rehabil, 34(8), 671-680. https://doi.org/10.3109/09638288.2011.619616

104. Nieuwkerk, P. T., Tollenaar, M. S., Oort, F. J., & Sprangers, M. A. G. (2007). Are Retrospective Measures of Change in Quality of Life More Valid Than Prospective Measures? Medical Care, 45(3), 199-205. https://doi.org/10.1097/01.mlr.0000246613.49214.46

105. Nolte, S., Mierke, A., Fischer, H. F., & Rose, M. (2016). On the validity of measuring change over time in routine clinical assessment: a close examination of item-level response shifts in psychosomatic inpatients. Quality of Life Research, 25(6), 1339-1347. https://doi.org/10.1007/s11136-015-1123-3

106. Ong, C. W., Pierce, B. G., Klein, K. P., Hudson, C. C., Beard, C., & Björgvinsson, T. (2021). Longitudinal measurement invariance of the PHQ-9 and GAD-7. Assessment, 29(8), 1901-1916. https://doi.org/10.1177/10731911211035833

107. Oort Frans, J., Visser Mechteld, R. M., & Sprangers Mirjam, A. G. (2005). An application of structural equation modeling to detect response shifts and true change in quality of life data from cancer patients undergoing invasive surgery. Quality of life research, 14(3), 599-609.

108. Oreel, T. H., Nieuwkerk, P. T., Hartog, I. D., Netjes, J. E., Vonk, A. B. A., Lemkes, J., van Laarhoven, H. W. M., Scherer-Rath, M., Henriques, J. P. S., Oort, F. J., Sprangers, M. A. G., & Verdam, M. G. E. (2021). Response shift after coronary revascularization. Quality of Life Research, 31(2), 437-450. https://doi.org/10.1007/s11136-021-02902-5

109. Ousmen, A., Conroy, T., Guillemin, F., Velten, M., Jolly, D., Mercier, M., Causeret, S., Cuisenier, J., Graesslin, O., Hamidou, Z., Bonnetain, F., & Anota, A. (2016). Impact of the occurrence of a response shift on the determination of the minimal important difference in a health-related quality of life score over time. Health and Quality of Life Outcomes, 14(1), 167. https://doi.org/10.1186/s12955-016-0569-5

110. Perdomo-Arguello, F. J., Ortega-Gomez, E., Galindo-Villardon, P., Leiva, V., & Vicente-Galindo, P. (2023). STATIS multivariate three-way method for evaluating quality of life after corneal surgery: Methodology and case study in Costa Rica. Mathematical Biosciences and Engineering, 20(4), 6110-6133. https://doi.org/10.3934/mbe.2023264

111. Persson, L. O., Engstrom, C. P., Ryden, A., Larsson, S., & Sullivan, M. (2005). Life values in patients with COPD: relations with pulmonary functioning and health related quality of life. Quality of Life Research, 14(2), 349-359. https://doi.org/10.1007/s11136-004-0623-3

112. Postulart, D., & Adang, E. M. M. (2016). Response shift and adaptation in chronically iII patients. Medical Decision Making, 20(2), 186-193. https://doi.org/10.1177/0272989x0002000204

113. Powell, G. A., Adair, C. E., Streiner, D. L., Mayo, N., & Latimer, E. (2017). Changes in quality of life from a homelessness intervention: true change, response shift, or random variation. Quality of Life Research, 26(7), 1853-1864. https://doi.org/10.1007/s11136-017-1522-8

114. Pratt, C. C., McGuigan, W. M., & Katzev, A. R. (2016). Measuring program outcomes: Using retrospective pretest methodology. American Journal of Evaluation, 21(3), 341-349. https://doi.org/10.1177/109821400002100305

115. Preiss, M., Friedrich, M., Stolzenburg, J. U., Zenger, M., & Hinz, A. (2019). Response shift effects in the assessment of urologic cancer patients' quality of life. European Journal Cancer Care, 28(4), e13027. https://doi.org/10.1111/ecc.13027

116. Rapkin Bruce, D. (2000). Personal goals and response shifts: Understanding the impact of illness and events on the quality of life of people living with AIDS. Adaptation to changing health: Response shift in quality-of-life research., 53-71. https://doi.org/http://dx.doi.org/10.1037/10382-004

117. Razmjou, H., Schwartz, C. E., & Holtby, R. (2010). The impact of response shift on perceived disability two years following rotator cuff surgery. The Journal of Bone and Joint Surgery, 92(12), 2178-2186. https://doi.org/92/12/2178

118. Razmjou, H., Schwartz, C. E., Yee, A., & Finkelstein, J. A. (2009). Traditional assessment of health outcome following total knee arthroplasty was confounded by response shift phenomenon. Journal of Clinical Epidemiology, 62(1), 91-96. https://doi.org/10.1016/j.jclinepi.2008.08.004

119. Razmjou, H., Yee, A., Ford, M., & Finkelstein, J. A. (2006). Response shift in outcome assessment in patients undergoing total knee arthroplasty. The Journal of Bone and Joint Surgery, 88(12), 2590-2595. https://doi.org/88/12/2590

120. Rees, J., Clarke Michael, G., Waldron, D., O'Boyle, C., Ewings, P., & MacDonagh Ruaraidh, P. (2005). The measurement of response shift in patients with advanced prostate cancer and their partners. Health and Quality of Life Outcomes, 3, 21.

121. Rees, J., Waldron, D., O'Boyle, C., Ewings, P., & MacDonagh, R. (2003). Prospective vs retrospective assessment of lower urinary tract symptoms in patients with advanced prostate cancer: the effect of 'response shift'. BJU Int, 92(7), 703-706. https://doi.org/4462

122. Reissmann, D. R., Erler, A., Hirsch, C., Sierwald, I., Machuca, C., & Schierz, O. (2017). Bias in retrospective assessment of perceived dental treatment effects when using the Oral Health Impact Profile. Quality of Life Research, 27(3), 775-782. https://doi.org/10.1007/s11136-017-1725-z

123. Reissmann, D. R., John, M. T., Feuerstahler, L., Baba, K., Szabó, G., Čelebić, A., & Waller, N. (2016). Longitudinal measurement invariance in prospective oral health-related quality of life assessment. Health and Quality of Life Outcomes, 14(1), 88. https://doi.org/10.1186/s12955-016-0492-9

124. Reissmann, D. R., Remmler, A., John, M. T., Schierz, O., & Hirsch, C. (2012). Impact of response shift on the assessment of treatment effects using the Oral Health Impact Profile. European Journal of Oral Sciences, 120(6), 520-525. https://doi.org/10.1111/j.1600-0722.2012.00998.x

125. Ring, L., Hofer, S., Heuston, F., Harris, D., & O'Boyle, C. A. (2005). Response shift masks the treatment impact on patient reported outcomes (PROs): the example of individual quality of life in edentulous patients. Health and Quality of Life Outcomes, 3, 55. https://doi.org/1477-7525-3-55

126. Rissanen, R., Warnqvist, A., & Hasselberg, M. (2023). “I knew who I was this morning, but I've changed a few times since then”: A study combining register and self-reported QoL data in assessing how response shift may occur in an injury population. Social Science & Medicine, 326, N.PAG-N.PAG. https://doi.org/10.1016/j.socscimed.2023.115916

127. Rosenström, T. H., Ritola, V., Saarni, S., Joffe, G., & Stenberg, J.-H. (2021). Measurement invariant but non-normal treatment responses in guided internet psychotherapies for depressive and generalized anxiety disorders. Assessment, 30(3), 618-632. https://doi.org/10.1177/10731911211062500

128. Rutgers, M., Creemers, L. B., Yang, K. G. A., Raijmakers, N. J. H., Dhert, W. J. A., & Saris, D. B. F. (2014). Osteoarthritis treatment using autologous conditioned serum after placebo. Acta Orthopaedica, 86(1), 114-118. https://doi.org/10.3109/17453674.2014.950467

129. Sajobi, T. T., Fiest, K. M., & Wiebe, S. (2014). Changes in quality of life after epilepsy surgery: The role of reprioritization response shift. Epilepsia, 55(9), 1331-1338. https://doi.org/10.1111/epi.12697

130. Sajobi, T. T., Lix, L. M., Singh, G., Lowerison, M., Engbers, J., & Mayo, N. E. (2015). Identifying reprioritization response shift in a stroke caregiver population: a comparison of missing data methods. Quality of Life Research, 24(3), 529-540. https://doi.org/10.1007/s11136-014-0824-3

131. Sajobi, T. T., Speechley, K. N., Liang, Z., Goodwin, S. W., Ferro, M. A., & Wiebe, S. (2017). Response shift in parents' assessment of health-related quality of life of children with new-onset epilepsy. Epilepsy & Behavior, 75, 97-101. https://doi.org/10.1016/j.yebeh.2017.07.015

132. Salmon, M., Blanchin, M., Rotonda, C., Guillemin, F., & Sébille, V. (2017). Identifying patterns of adaptation in breast cancer patients with cancer‐related fatigue using response shift analyses at subgroup level. Cancer Medicine, 6(11), 2562-2575. https://doi.org/10.1002/cam4.1219

133. Schwartz, C. E., Feinberg, R. G., Jilinskaia, E., & Applegate, J. C. (1999). An evaluation of a psychosocial intervention for survivors of childhood cancer: paradoxical effects of response shift over time. Psycho-Oncology, 8(4), 344-354. https://doi.org/10.1002/(SICI)1099-1611(199907/08)8:4<344::AID-PON399>3.0.CO;2-T

134. Schwartz, C. E., Merriman, M. P., Reed, G. W., & Hammes, B. J. (2004). Measuring patient treatment preferences in end-of-life care research: applications for advance care planning interventions and response shift research. Journal of Palliative Medicine, 7(2), 233-245. https://doi.org/10.1089/109662104773709350

135. Schwartz, C. E., Powell, V. E., & Rapkin, B. D. (2016). 23rd Annual Conference of the International Society for Quality of Life Research. Quality of Life Research, 25(S1), 1-196. https://doi.org/10.1007/s11136-016-1390-7

136. Schwartz, C. E., & Rapkin, B. D. (2012). Understanding appraisal processes underlying the thentest: a mixed methods investigation. Quality of Life Research, 21(3), 381-388. https://doi.org/10.1007/s11136-011-0023-4

137. Schwartz, C. E., Sajobi, T. T., Lix, L. M., Quaranto, B. R., & Finkelstein, J. A. (2013). Changing values, changing outcomes: the influence of reprioritization response shift on outcome assessment after spine surgery. Quality of Life Research, 22(9), 2255-2264. https://doi.org/10.1007/s11136-013-0377-x

138. Schwartz, C. E., Sprangers, M. A. G., Carey, A., & Reed, G. (2004). Exploring response shift in longitudinal data. Psychology & Health, 19(1), 51-69.

139. Schwartz, C. E., Stark, R. B., Borowiec, K., Nolte, S., & Myren, K. J. (2021). Norm-based comparison of the quality-of-life impact of ravulizumab and eculizumab in paroxysmal nocturnal hemoglobinuria. Orphanet J Rare Dis, 16(1), 389. https://doi.org/10.1186/s13023-021-02016-8

140. Schwartz, C. E., Stark, R. B., & Stucky, B. D. (2021). Response-shift effects in neuromyelitis optica spectrum disorder: a secondary analysis of clinical trial data. Quality of Life Research, 30(5), 1267-1282. https://doi.org/10.1007/s11136-020-02707-y

141. Schwartz, C. E., Stark, R. B., & Stucky, B. D. (2021). Response-shift effects in neuromyelitis optica spectrum disorder: a secondary analysis of clinical trial data. Quality of Life Research, 30(5), 1267-1282. https://doi.org/10.1007/s11136-020-02707-y

142. Schwartz, C. E., Stark, R. B., Stucky, B. D., Li, Y., & Rapkin, B. D. (2021). Response-shift effects in neuromyelitis optica spectrum disorder: estimating response-shift-adjusted scores using equating. Quality of Life Research, 30(5), 1283-1292. https://doi.org/10.1007/s11136-020-02727-8

143. Schwartz, C. E., Stucky, B., Rivers, C. S., Noonan, V. K., & Finkelstein, J. A. (2018). Quality of life and adaptation in people with spinal cord injury: response shift effects from 1 to 5 years postinjury. Archives of Physical Medicine and Rehabilitation, 99(8), 1599-1608 e1591. https://doi.org/S0003-9993(18)30117-5

144. Schwartz, C. E., Stucky, B. D., Michael, W., & Rapkin, B. D. (2020). Does response shift impact interpretation of change even among scales developed using item response theory? Journal of Patient-Reported Outcomes, 4(1), 8. https://doi.org/10.1186/s41687-019-0162-x

145. Serdà i Ferrer, B.-C., Valle, A. d., & Marcos-Gragera, R. (2014). Prostate cancer and quality of life: analysis of response shift using triangulation between methods. Journal of Gerontological Nursing, 40(6), 32-41. https://doi.org/10.3928/00989134-20140211-01

146. Sharpe, L., Butow, P., Smith, C., McConnell, D., & Clarke, S. (2005). Changes in quality of life in patients with advanced cancer: evidence of response shift and response restriction. Journal of Psychosomatic Research, 58(6), 497-504. https://doi.org/S0022-3999(05)00077-2

147. Shi, H. Y., Lee, K. T., Lee, H. H., Uen, Y. H., & Chiu, C. C. (2011). Response shift effect on gastrointestinal quality of life index after laparoscopic cholecystectomy. Quality of Life Research, 20(3), 335-341. https://doi.org/10.1007/s11136-010-9760-z

148. Smith, D., Woodman, R., Harvey, P., & Battersby, M. (2016). Self-perceived distress and impairment in problem gamblers: a study of pre- to post-treatment measurement invariance. Journal of Gambling Studies, 32(4), 1065-1078. https://doi.org/10.1007/s10899-016-9598-6

149. Sprangers, M. A. (1996). Response-shift bias: a challenge to the assessment of patients' quality of life in cancer clinical trials. Cancer Treatment Reviews, 22 Suppl A, 55-62. https://doi.org/S0305-7372(96)90064-X

150. Sprangers Mirjam, A. G., van, D., Frits, S. A. M., Broersen, J., Lodder, L., Wever, L., Visser Mechteld, R. M., Oosterveld, P., & Smets, E. (2000). Response shift and fatigue: The use of the thentest approach. Adaptation to changing health: Response shift in quality-of-life research., 137-151. https://doi.org/http://dx.doi.org/10.1037/10382-009

151. Spuling, S. M., Wolff, J. K., & Wurm, S. (2017). Response shift in self-rated health after serious health events in old age. Social Science & Medicine, 192, 85-93. https://doi.org/S0277-9536(17)30562-2

152. Svedbom, A., Borgstom, F., Hernlund, E., Alekna, V., Bianchi, M. L., Clark, P., Diaz-Curiel, M., Dimai, H. P., Jurisson, M., Lesnyak, O., McCloskey, E., Sanders, K. M., Silverman, S., Tamulaitiene, M., Thomas, T., Tosteson, A. N. A., Jonsson, B., & Kanis, J. A. (2023). An experience- and preference-based EQ-5D-3L value set derived using 18 months of longitudinal data in patients who sustained a fracture: results from the ICUROS. Quality of Life Research, 32(4), 1199-1208. https://doi.org/10.1007/s11136-022-03303-y

153. ten Ham, R. M. T., Broering, J. M., Cooperberg, M. R., Carroll, P., & Wilson, L. S. (2020). Understanding the major factors affecting response shift effects on health-related quality of life: What the then-test measures in a longitudinal prostate cancer registry. Clinical Genitourinary Cancer, 18(1), e21-e27. https://doi.org/10.1016/j.clgc.2019.10.026

154. Tessier, P., Blanchin, M., & Sébille, V. (2017). Does the relationship between health-related quality of life and subjective well-being change over time? An exploratory study among breast cancer patients. Social Science & Medicine, 174, 96-103. https://doi.org/10.1016/j.socscimed.2016.12.021

155. Testa, S., Di Cuonzo, D., Ritorto, G., Fanchini, L., Bustreo, S., Racca, P., & Rosato, R. (2021). Response shift in health-related quality of life measures in the presence of formative indicators. Health and Quality of Life Outcomes, 19(1), 9. https://doi.org/10.1186/s12955-020-01663-y

156. Timmerman Angelique, A., Anteunis Lucien, J. C., & Meesters Cor, M. G. (2003). Response-shift bias and parent-reported quality of life in children with otitis media. Archives of Otolaryngology--Head & Neck Surgery, 129(9), 987-991.

157. Traa, M. J., Braeken, J., De Vries, J., Roukema, J. A., Orsini, R. G., & Den Oudsten, B. L. (2014). Evaluating quality of life and response shift from a couple-based perspective: a study among patients with colorectal cancer and their partners. Quality of Life Research, 24(6), 1431-1441. https://doi.org/10.1007/s11136-014-0872-8

158. van Leeuwen, C. M. C., Post, M. W. M., van der Woude, L. H. V., de Groot, S., Smit, C., van Kuppevelt, D., & Lindeman, E. (2011). Changes in life satisfaction in persons with spinal cord injury during and after inpatient rehabilitation: adaptation or measurement bias? Quality of Life Research, 21(9), 1499-1508. https://doi.org/10.1007/s11136-011-0073-7

159. Verdam, M. G., Oort, F. J., & Sprangers, M. A. (2016). Using structural equation modeling to detect response shifts and true change in discrete variables: an application to the items of the SF-36. Quality of Life Research, 25(6), 1361-1383. https://doi.org/10.1007/s11136-015-1195-0

160. Verdam, M. G. E., Oort, F. J., & Sprangers, M. A. G. (2017). Structural equation modeling–based effect-size indices were used to evaluate and interpret the impact of response shift effects. Journal of Clinical Epidemiology, 85, 37-44. https://doi.org/10.1016/j.jclinepi.2017.02.012

161. Verdam, M. G. E., Oort, F. J., van der Linden, Y. M., & Sprangers, M. A. G. (2014). Taking into account the impact of attrition on the assessment of response shift and true change: a multigroup structural equation modeling approach. Quality of Life Research, 24(3), 541-551. https://doi.org/10.1007/s11136-014-0829-y

162. Verdam, M. G. E., van Ballegooijen, W., Holtmaat, C. J. M., Knoop, H., Lancee, J., Oort, F. J., Riper, H., van Straten, A., Verdonck-de Leeuw, I. M., de Wit, M., van der Zweerde, T., & Sprangers, M. A. G. (2021). Re-evaluating randomized clinical trials of psychological interventions: Impact of response shift on the interpretation of trial results. PLoS One, 16(5), e0252035. https://doi.org/PONE-D-21-02214

163. Visser, M. R., Oort, F. J., & Sprangers, M. A. (2005). Methods to detect response shift in quality of life data: a convergent validity study. Quality of Life Research, 14(3), 629-639. https://doi.org/10.1007/s11136-004-2577-x

164. Visser, M. R., Smets, E. M., Sprangers, M. A., de, H., & H, J. (2000). How response shift may affect the measurement of change in fatigue. Journal of Pain and Symptom Management, 20(1), 12-18.

165. Visser, M. R. M., Oort, F. J., van Lanschot, J. J. B., van der Velden, J., Kloek, J. J., Gouma, D. J., Schwartz, C. E., & Sprangers, M. A. G. (2012). The role of recalibration response shift in explaining bodily pain in cancer patients undergoing invasive surgery: an empirical investigation of the Sprangers and Schwartz model. Psycho-Oncology, 22(3), 515-522. https://doi.org/10.1002/pon.2114

166. Wagner Julie, A. (2005). Response shift and glycemic control in children with diabetes. Health and Quality of Life Outcomes, 3, 38.

167. Wang, X., Xu, X., Han, H., He, R., Zhou, L., Liang, R., & Yu, H. (2019). Using structural equation modeling to detect response shift in quality of life in patients with Alzheimer's disease. International Psychogeriatrics, 31(1), 123-132. https://doi.org/S1041610218000595

168. Wu, P. C. (2016). Response shifts in depression intervention for early adolescents. Journal of Clinical Psychology, 72(7), 663-675. https://doi.org/10.1002/jclp.22291

169. Wu, Q., & Zhang, P. (2023). Longitudinal validity of self-rated health: the presence and impact of response shift. Psychology & Health, 38(7), 905-926. https://doi.org/10.1080/08870446.2021.1994571

170. Yang, J., Hanna-Pladdy, B., Gruber-Baldini, A. L., Barr, E., von Coelln, R., Armstrong, M. J., Reich, S. G., & Shulman, L. M. (2017). Response shift - The experience of disease progression in Parkinson disease. Parkinsonism Relat Disord, 36, 52-56. https://doi.org/S1353-8020(16)30520-X

171. Yardley, L., & Dibb, B. (2007). Assessing subjective change in chronic illness: an examination of response shift in health-related and goal-oriented subjective status. Psychology & Health, 22(7), 813-828.

172. Zeldovich, M., Hahm, S., Mueller, I., Krenz, U., Bockhop, F., von Steinbuechel, N., The Center-Tbi, P., & Investigators. (2023). Longitudinal internal validity of the quality of life after brain injury: response shift and responsiveness. Journal of Clinical Medicine, 12(9). https://doi.org/jcm12093197

173. Zhang, X.-H., Li, S.-C., Xie, F., Lo, N.-N., Yang, K.-Y., Yeo, S.-J., Fong, K.-Y., & Thumboo, J. (2012). An exploratory study of response shift in health-related quality of life and utility assessment among patients with osteoarthritis undergoing total knee replacement surgery in a tertiary hospital in Singapore. Value in Health, 15(1), S72-S78. https://doi.org/10.1016/j.jval.2011.11.011
